# Non-Genomic Actions of Testosterone Metabolites on Uterine Contractility in Rats

**DOI:** 10.3390/pharmaceutics18091063

**Published:** 2026-08-26

**Authors:** Saif-alnasr H. Mohammed, Ayman B. Mousa, Mohammed Taj-Eldin Abdalla, Anita Sztojkov-Ivanov, Kálmán F. Szűcs, Róbert Gáspár

**Affiliations:** 1 Department of Pharmacology and Pharmacotherapy, Albert-Szent-Györgyi Medical School, University of Szeged, 6720 Szeged, Hungary; saif-alnasr.hassan.mohammed@med.u-szeged.hu (S.-a.H.M.); ayman.bakheet.musa.mohammed@med.u-szeged.hu (A.B.M.); mohammed.taj@med.u-szeged.hu (M.T.-E.A.); szucs.kalman@med.u-szeged.hu (K.F.S.); gaspar.robert@med.u-szeged.hu (R.G.); 2 Department of Pharmacology, Faculty of Pharmacy, Omdurman Islamic University, Omdurman 14415, Sudan; 3 Department of Clinical Pharmacology, Faculty of Medicine, University of Bahri, Alkadroo 11111, Sudan; 4 Department of Pharmacology and Pharmacy Practice, Faculty of Pharmacy, Sudan University of Science and Technology, Khartoum 11111, Sudan; 5 Department of Pharmacodynamics and Biopharmacy, Faculty of Pharmacy, University of Szeged, 6720 Szeged, Hungary; sztojkov.ivanov.anita@szte.hu

**Keywords:** non-genomic action, 5α-dihydrotestosterone, 5β-dihydrotestosterone, pharmacokinetic, pregnant rats, sex hormone, in vitro action, in vivo action

## Abstract

**Background**: Sex hormones play crucial functions in the body via the genomic and non-genomic pathways. 5α- and 5β-dihydrotestosterone (5α- and 5β-DHT) are 5-reduced testosterone metabolites. We aimed to investigate the non-genomic effect of 5α- and 5β-DHT on uterine muscle contractility in vitro and in vivo for non-pregnant and 22-day-pregnant rats. **Methods**: The rapid in vitro action of 5α-DHT and 5β-DHT (10^−9^–10^−3^ M) on KCl (25 mM)-stimulated contractions was examined in an organ bath in the presence of several blockers and after endometrium removal. The actions of DHTs (10^−4^ M) and nifedipine (10^−7^ M) were also examined in contractions stimulated by KCl (40 mM) with a cumulative addition of CaCl2 (3–120 mM). Plasma DHT levels were measured by ELISA after a single intraperitoneal (i.p.) administration of DHT (10 mg/kg), and kinetic curves were obtained. The in vivo relaxing action of DHTs was detected by strain-gauge sensors. The animals received 5α- or 5β-DHT alone (3/10/30/100/300 mg/kg i.p.) or with flutamide (100 mg/kg i.p.). **Results**: DHT showed concentration-dependent relaxation of uterine muscle in vitro, with a higher potency observed for 5β-DHT. Among the blockers used, G15 and L-NAME reduced the potency of 5α-DHT in pregnant rats only. Both DHTs inhibited the contraction-increasing effect of CaCl_2_, proving their Ca^2+^ inhibiting effects. DHTs had similar c_max_ and t_max_ values in both non-pregnant and pregnant rats. DHT plasma levels before and 30 min after administration were proportional to the administered doses. Their single doses (30/100/300 mg/kg) elicited a flutamide-resistant uterine relaxing effect in vivo. **Conclusions**: DHTs or their analogs are candidates for further studies in the human uterus to establish their potential to treat conditions associated with uterine hyperactivity.

## 1. Introduction

Androgens are sex steroids that play important functions in the body, particularly in the development [1,2,3,4,5], metabolism [1,6], reproduction [3,4,5], hemostasis [4], and modulation of various muscle tones, especially in the cardiovascular [7,8], airway [9] and uterine muscles [3,10,11]. These activities are attributed to interaction with the nuclear androgen receptor (AR), which initiates a cascade of gene expression called “genomic actions”; however, they also elicit non-transcriptional responses, referred to as “non-genomic actions” [3,10,12].

5α- and 5β-dihydrotestosterones (5α- and 5β-DHT) are metabolites of testosterone (T) [13,14]. Most of 5α-DHT is formed via the conversion of testosterone [15], and a little is produced directly from the testis or adrenal gland, or converted from progestin [14,16]. DHTs are metabolized to inactive metabolites via 3α-17β-hydroxysteroid dehydrogenase (3α-HSD) and 3β-17β-hydroxysteroid dehydrogenase (3β-HSD), and the metabolites are conjugated by uridine 5’-diphospho-glucuronosyltransferase (UGT) to be excreted via urinary and biliary elimination. DHTs cannot be converted to estrogens, unlike T [15,17].

The plasma level of 5α-DHT is thought to be 10% of that of T [14,18]. It exerts more androgenic nuclear action with a longer half-life than T, as well as higher affinity and slower dissociation, resulting in higher potency [14,15,19]. 5β-DHT has received less attention, and only limited information is available about it; it is believed to have no androgenic effects. There is no data available on the conversion ratio of T to 5α- or 5β-DHT; nevertheless, the proportion of expression of 5α- and 5β-reductase enzymes varies according to tissue type [20,21,22]. In the liver, the 5β-reductase enzyme participates in the inactivation of androgens, as many studies suggest that 5β-DHT is an inactive metabolite in terms of androgenic activity. It is also expressed in the testicles and is involved in bile acid biosynthesis [22,23]. However, 5β-DHT was shown to initiate a rapid smooth muscle relaxant effect in human umbilical arteries [18] and rat vascular smooth muscle [13]. Also, 5α-DHT shows a relaxing effect on guinea pig gallbladder strips and rat blood vessels via a non-nuclear mechanism [13,19].

The non-genomic effects are characterized by a rapid initiation of action; the time gap between drug administration and the onset of action is less than 30 min. The mechanism might involve the stimulation of MAPK and ERK1/2 signaling pathways, together with protein kinase A and C pathways [10,24,25]. Membrane proteins such as G-protein-coupled receptors (GPCRs), enzyme-linked receptors, and ion channels also participate in non-genomic signaling [7,10,26]. Both G-protein-coupled receptor class C, group 6, subtype A (GPCRC6A) and Zinc Transporter 9.(ZIP9) are known as targets of androgens [4,12,27].

Previous in vitro studies demonstrated that T induced a non-genomic uterine muscle relaxant action in a concentration-dependent manner that was resistant to androgen blocker (flutamide), protein synthesis and transcription inhibitors (cycloheximide and actinomycin D, respectively) [3,11]. Additionally, T was found to relax uterine smooth muscles in a dose-dependent manner via a non-genomic pathway [28]. The T metabolites, 5α- and 5β-DHT, show more potent actions than T itself [11,14]; however, their non-genomic effects on uterine muscle contractility have not yet been investigated.

Although the non-genomic effects of DHT have been demonstrated in numerous types of smooth muscle—including vascular and visceral smooth muscle—the direct effects of 5α- and 5β-DHT on uterine smooth muscle remain unclear, particularly during pregnancy. The mechanisms of action are unknown, as is whether the two isomers exert similar or different effects. Elucidating these mechanisms could pave the way for the clinical use of DHT in cases of excessive uterine contractility, such as in the event of threatened preterm labor.

Therefore, we aimed to investigate the non-genomic effect of 5α- and 5β-DHT on uterine muscle contractility in vitro and in vivo for non-pregnant and 22-day-pregnant rats and their underlying mechanism of action.

## 2. Materials and Methods

### 2.1. Drugs and Chemicals

5α-DHT, flutamide, mifepristone, paxilline and calindol were purchased from Sigma-Aldrich (Budapest, Hungary); 5β-DHT was purchased from LinkChem Co., Ltd. (Shanghai, China); NPS-2143 and G15 were purchased from AdooQ Bioscience (Irvine, CA, USA); N(ω)-nitro-L-arginine methyl ester (L-NAME) was purchased from MedChemExpress (Township, NJ, USA); dimethyl sulfoxide (DMSO) was from Fisher Scientific (Loughborough, UK); macrogol 400 was purchased from MAGIlab Ltd. (Budapest, Hungary); and rat DHT, cyclic adenosine monophosphate (cAMP) and cyclic guanosine monophosphate (cGMP) enzyme-linked immunosorbent assay (ELISA) kits were delivered by Wuhan Fine Biotech Co., Ltd. (Wuhan, China).

### 2.2. Housing and Handling of Animals

Sprague Dawley (SPRD) female rats (160–220 g) were chosen for the experiment; they were housed in the animal facility of the Department of Pharmacology and Pharmacotherapy, Albert Szent-Györgyi Medical School, University of Szeged under controlled temperature, humidity and light (20–23 °C, 40–60% and 12 h light/dark cycle, respectively). The animals were kept on a standard Altromin 1324 rodent pellet diet (Charles-River Laboratories, Sulzfeld, Germany), with tap water available ad libitum.

### 2.3. Mating and Selection of Rats

The mature female rats in the estrus cycle were chosen based on vaginal impedance with an Estrus Cycle Monitor (IM-01, MSB-MET Ltd., Balatonfüred, Hungary). Rats whose vaginal impedance on the day of the experiment was 4.5–7.5 kΩ were chosen for non-pregnant or mating experiments. For mating, SPRD male rats (240–260 g) were placed separately in a mating cage divided into 2 compartments by an automated movable metal gate. The gate was pulled up at 4 a.m., and mating was possible in 4 to 5 h. To confirm intercourse, native vaginal smears or copulation plugs were checked under the microscope at 1200× magnification. In the case of spermatozoa present in the sample or a sperm plug visible in the vagina, pregnancy was confirmed and the day of copulation was designated as the first day of pregnancy. Positive cases were housed in separate cages and used on the 22nd day of pregnancy. Animals that did not meet the predetermined requirements regarding body weight, estrous cycle, and confirmation of pregnancy were not included in the appropriate experimental groups.

Considering the 3Rs, the minimum number of experimental animals was calculated using statistical software G*Power 3.1.9.2, based on α = 0.01 and 0.8 power settings and assuming equal numbers of control and treated individuals. All estrus or pregnant rats were selected and simultaneously randomized to the groups using a computer-based random order generator, and then the position of the cages was also randomized. The investigators could not be blinded to the groups and treatments of rats, but all animals in the experiment were handled, anesthetized, and treated in the same way. No enrolled animals were excluded after allocation into experimental groups.

### 2.4. Selection of DHT Concentration and Doses

For in vitro experiments, the concentration ranges of 5α- and 5β-DHT (10^−9^ to 10^−3^ M) were selected based on the values previously used for T [3]. The in vivo dose range was selected based on the literature on systemic administration of T in male [29] and female rats [28]. As 5α- and 5β-DHT are T metabolites, the same concentration and dose range was used, which is expected to produce a comparable effect.

### 2.5. Study of Isolated Organ Baths

**Figure 1:**
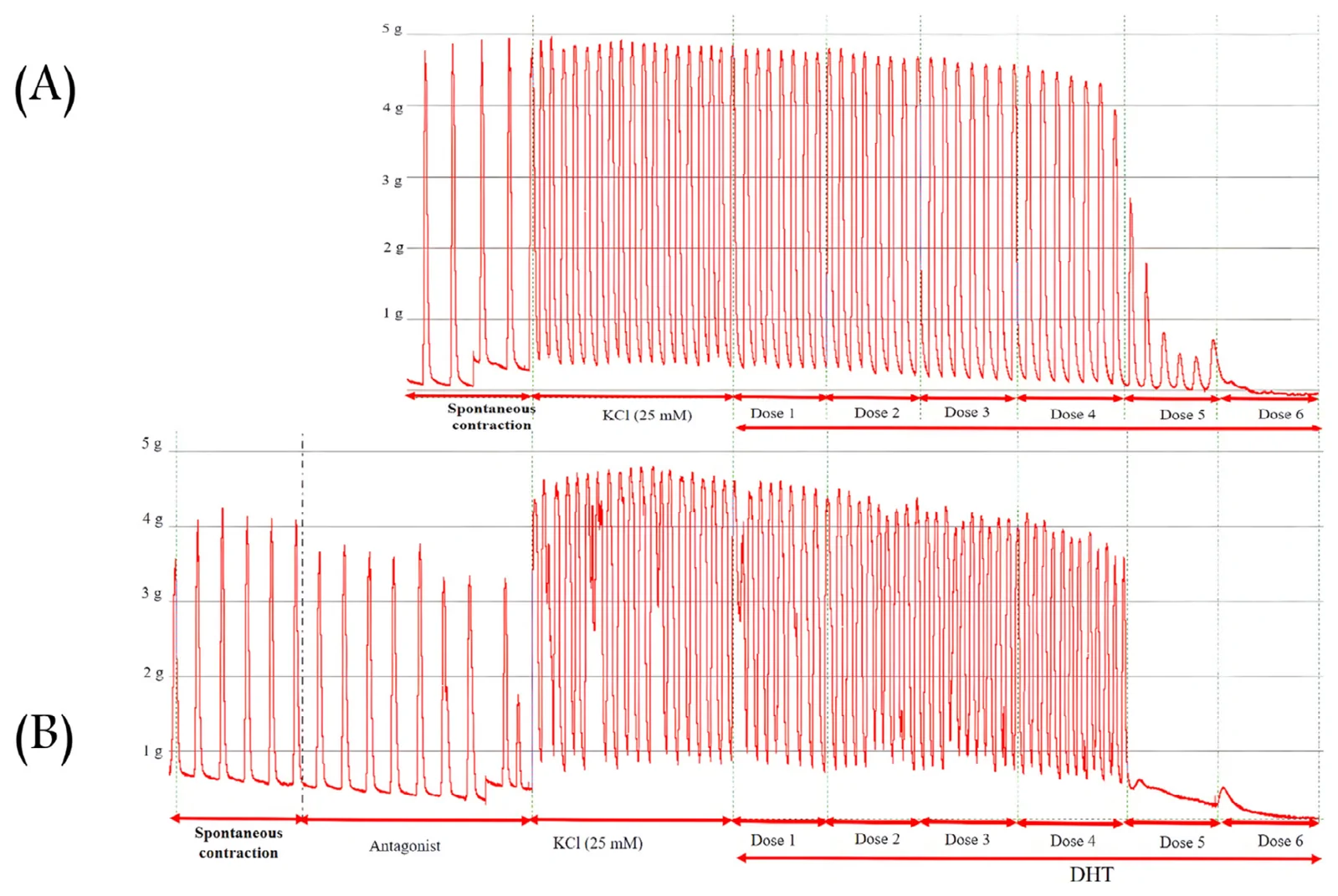
Representative records for the effects of 5α-DHT (**A**) and flutamide (antagonist) + 5α-DHT (**B**) on uterine muscles in vitro.

**Figure 2:**
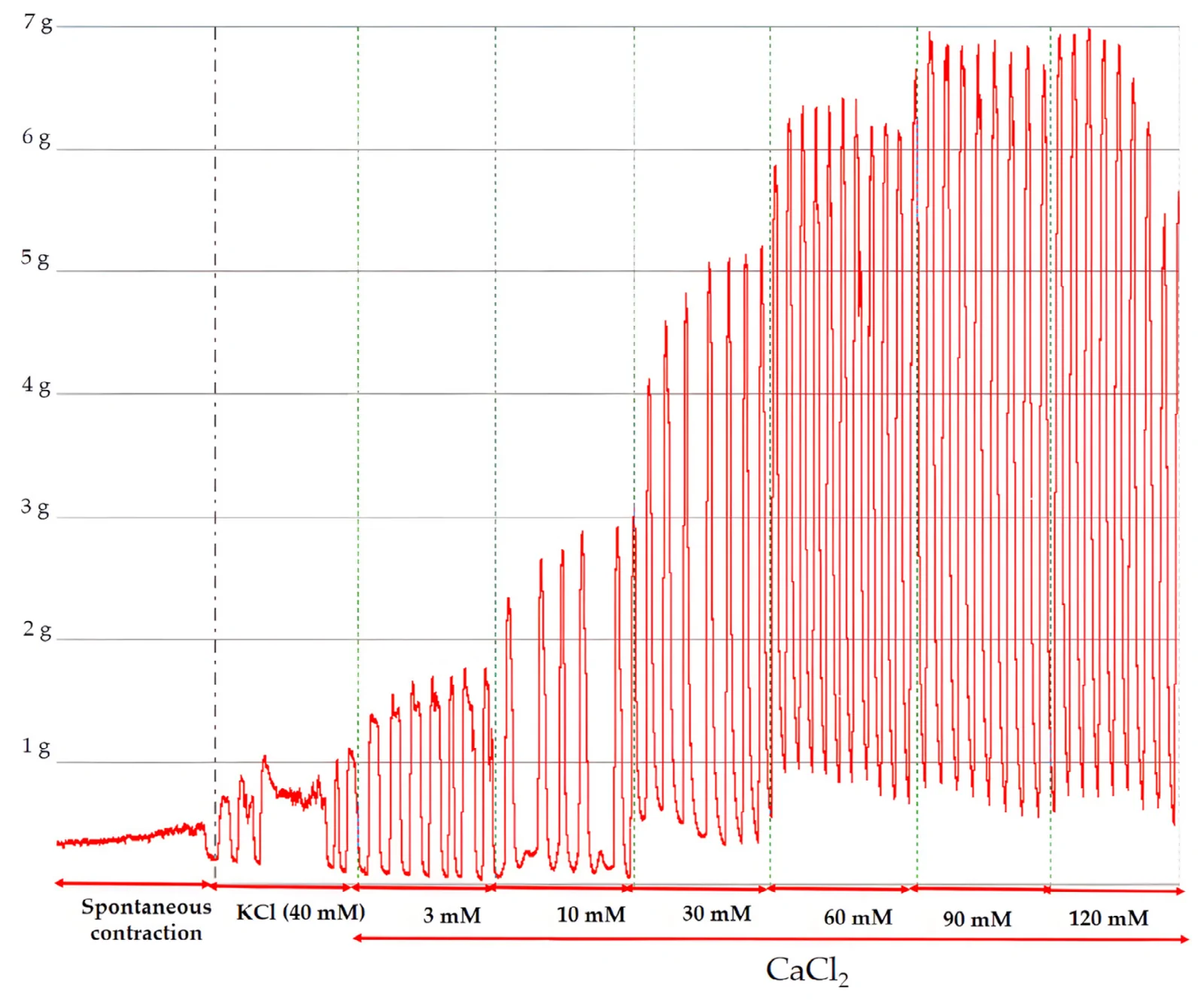
A representative record for the effects of cumulative addition of CaCl_2_ on KCl-induced uterine contractions in vitro.

The animals were sacrificed in a carbon dioxide chamber, and uterus samples were cut from both sides of the uterine horns. After cleaning of connective and adipose tissue, 3–4 mm dissected uterine tissues were tied with silk thread and vertically mounted in an isolated organ bath filled with 10 mL of de Jongh buffer (137 mM NaCl, 3 mM KCl, 1 mM CaCl_2_, 1 mM MgCl_2_, 12 mM NaHCO_3_, 4 mM NaH_2_PO_4_, 6 mM glucose). The pH was adjusted between 7.35 and 7.40 at a constant temperature (37 °C) and with carbogen (95% O_2_ + 5% CO_2_) support. The tissues were attached to a gauge transducer (SG-02; MSB-MET Ltd., Balatonfüred, Hungary), with an initial resting tension of 1.5 g; the contractions were measured, recorded and analyzed with an SPEL Advanced ISOSYS Data Acquisition System (MDE GmBH., Heidelberg, Germany). The tissues were washed every 15 min during the 45 min equilibrium incubation period. To achieve a satisfactory rhythmic contraction response, KCl (25 mM) was added to each chamber for 10 min. Each androgen metabolite was added cumulatively (5α-DHT 10^−8^–10^−3^ M or 5β-DHT; 10^−9^–10^−4^ M) at 5 min intervals. 5α- and 5β-DHT are lipid soluble; hence, for the in vitro experiments, they were dissolved in a solvent consisting of ethanol, macrogol and distilled water (1:5:4), and the solvent effect on muscle contraction was subtracted. In another set of experiments, uterine tissues were pretreated for 10-15 min before KCl stimulation with the AR antagonist flutamide (10^−6^ M); the selective zinc transporter 9 (ZIP9, a non-classical membrane AR) blocker bicalutamide (10^−6^ M); the progesterone and glucocorticoid receptor antagonist mifepristone (10^−6^ M); the G-protein estrogen receptor (GPER) blocker G15 (10^−6^ M); the large-conductance calcium-activated potassium (BK) channel blocker paxilline (10^−5^ M); the calcium sensing receptor (CaSR) antagonist NPS-2143 (10^−5^ M); the CaSR activator calindol (10^−5^ M); or the nitric oxide synthase (NOS) inhibitor N(ω)-nitro-L-arginine methyl ester (L-NAME) (10^−5^ M). Furthermore, the endometrium of the uterine tissues was removed by scraping, and the experiments were repeated to observe the effect of the DHTs on the myometrium. Concentration–response curves were plotted against the KCl-stimulated contraction response, and the effects of androgen metabolites were expressed as percentage change. Moreover, in another set of experiments and in a de Jongh calcium (Ca^+2^)-free buffer, KCl (40 mM) was added to each chamber for 5 min to achieve contraction response, then calcium chloride (CaCl_2_) was added in a cumulative way (3, 10, 30, 60, 90 and 120 mM) every 3 min. In another set, uterine tissues were pretreated with a single dose of 5α-DHT (10^−4^ M), 5β-DHT (10^−5^ M) or nifedipine (10^−7^ M) for 5 min before KCl stimulation (40 mM), followed by the addition of calcium chloride (CaCl_2_) in a cumulative way (3, 10, 30, 60, 90, and 120 mM). The contraction response percentage was calculated based on the KCl response. The concentration–response curves were compared in the presence and absence of a single addition of DHTs or nifedipine, and the effects were expressed as percentage change. The samples for each experiment were collected from both sides of the uterine horns of 2 animals (8 rings/experiment) and repeated 3 times for each individual set of experiments (n = 6/group) (Figure 1 and Figure 2). 

### 2.6. Cyclic AMP and Cyclic GMP Studies

Uterine tissue samples from non-pregnant and 22-day-pregnant SPRD rats (n = 4–6) were incubated in an organ bath filled with de Jongh buffer. The samples were incubated, and 25 mM KCl was added for 10 min. Then, 5α-DHT (10^−4^ M and 10^−3^ M), 5β-DHT (10^−5^ M and 10^−4^ M), or control (vehicle) was added for 5 min. In the case of cGMP measurements, L-NAME (10^−5^ M) was also added to the chambers. The samples were then snap-frozen in liquid nitrogen and stored at −80 °C until homogenization was carried out. Each sample was ground using a micro-dismembrator, then phosphate-buffered saline (PBS) (9 mL/g tissue) containing protease inhibitor cocktail (10 µL/mL) was added to each sample and subjected to ultrasonic disruption for 10 min. The homogenate was centrifuged for 10 min at 10,000× *g*, then the supernatant was collected and stored at −80 °C until the assay was performed. Cyclic AMP (cAMP) and cyclic GMP (cGMP) levels in uterine tissues were measured using commercial cAMP and cGMP ELISA Kits according to the manufacturer’s instructions.

### 2.7. Pharmacokinetic Analysis

Pharmacokinetic parameters after intraperitoneal (i.p.) administration of 5α-DHT or 5β-DHT were calculated for the time–concentration data using non-compartmental analysis by Phoenix WinNonlin Software (Version 8.5.2.4, Certara USA, Inc., Rador, PA, USA). Baseline correction for endogenous 5α-DHT or 5β-DHT level was performed by subtracting pre-dose (time zero) concentrations from all subsequent time points. The peak plasma concentration (c_max_) and the peak plasma concentration time (t_max_) were obtained from the plasma concentration versus time profiles. The elimination rate constant was estimated as the terminal slope (λZ) by performing a linear regression analysis on the terminal phase of the logarithmic concentration versus time curves. The area under the plasma concentration curve of zero to 480 min (AUC0-480 min) was determined by the linear log trapezoidal method. The area from zero to infinity (AUC0-inf) was calculated by extrapolating to infinity using the equation (AUC0-inf = AUC0-480 min + ct/λZ), where ct is the concentration measured at 480 min. The elimination half-life (t_½_) of the terminal elimination phase was estimated using the formula t_½_ = ln2/λZ. The mean residence time (MRT0-inf) was calculated with the formula MRT0-inf = AUMC0-inf/AUC0-inf, where AUMC0-inf is the area under the first moment curve extrapolated to infinity. Total body clearance for extravascular administration (Cl/F) and the volume of distribution based on the terminal phase (VZ/F) were determined using the equations Cl/F = Dose/AUC0-inf and VZ/F = Dose/(λZ·AUC0-inf), respectively, where F is the fraction of dose absorbed. All data reported are means ± standard deviation (SD).

### 2.8. Detection of Plasma Levels of Dihydrotestosterone (DHT)

Plasma DHT levels were measured before (baseline) and after i.p. administration of a single dose of 5α- or 5β-DHT (10 mg/kg) for non-pregnant and 22-day-pregnant rats (n = 5/group) using an ELISA kit with a detection range of 39.063–2500 pg/mL and sensitivity of 23.438 pg/mL. Blood samples (1 mL) were collected from the tail vein before and after administration of 5α- or 5β-DHT (0, 5, 15, 30, 60, 120, 240 and 480 min) in tubes (BD Microtainer, Thermo Fisher Scientific Inc., Budapest, Hungary) containing K2EDTA (1 mg/tube) and subsequently centrifuged (1700× *g*, 10 min, 4 °C) to isolate plasma. DHT levels were also measured before (baseline) and 30 min after administration of single 5α- or 5β-DHT doses (30, 100 or 300 mg/kg, i.p.) for non-pregnant and 22-day-pregnant rats. Plasma samples were stored at −80 °C until the testosterone assay was carried out according to the manufacturer’s instructions. The DHT level was expressed as plasma level (pg/mL), and the physiological baseline plasma values of DHT were excluded from the measured values. The ELISA kit was not selective for DHT stereoisomers; therefore, any change observed in DHT levels was considered to be related to the stereoisomer administered specifically.

### 2.9. In Vivo Contractility Studies

**Figure 3:**
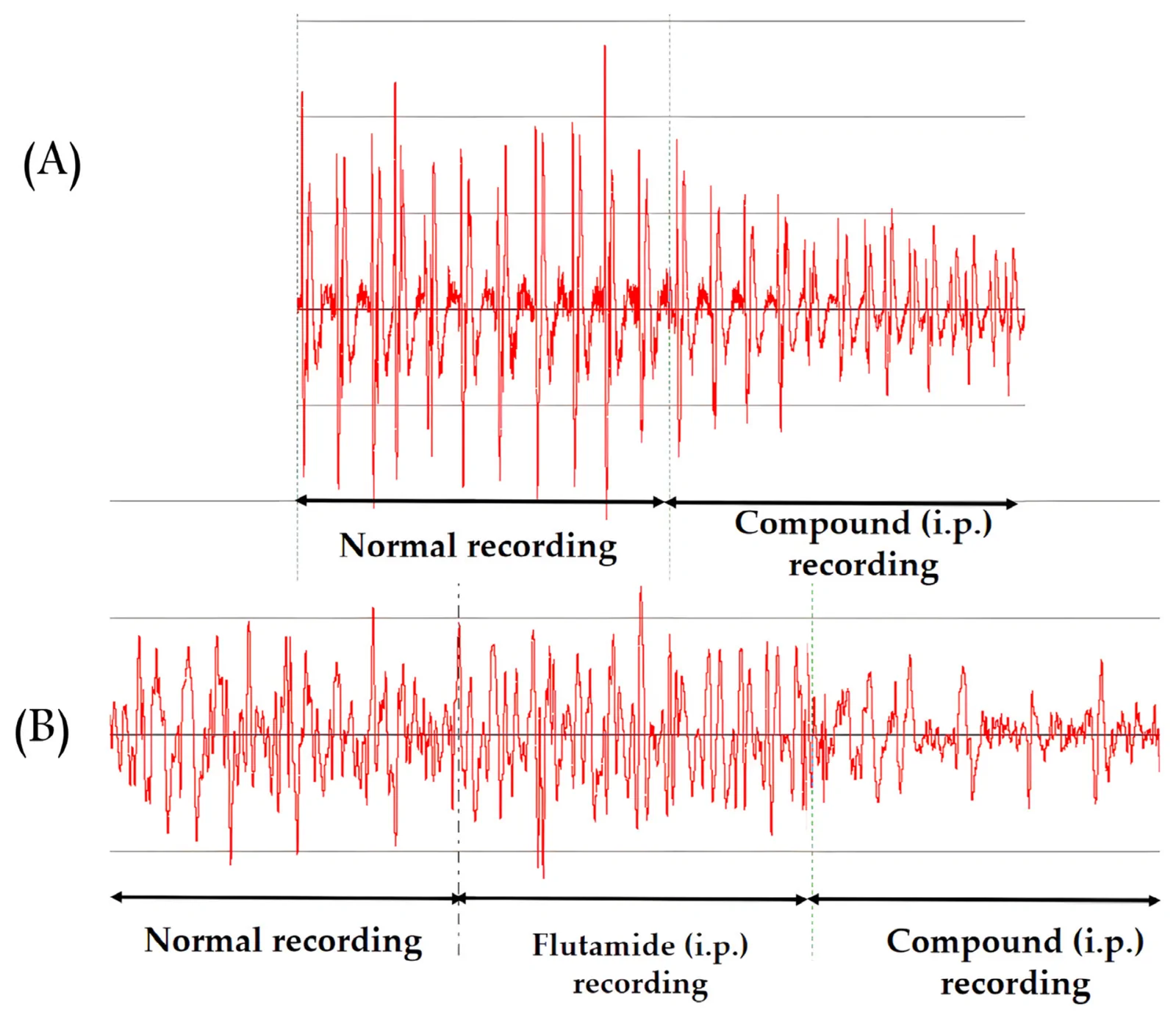
Representative records for the effects of 5*α*-DHT alone (**A**) and in the presence of flutamide (**B**) on the uterine muscles in vivo.

**Figure 4:**
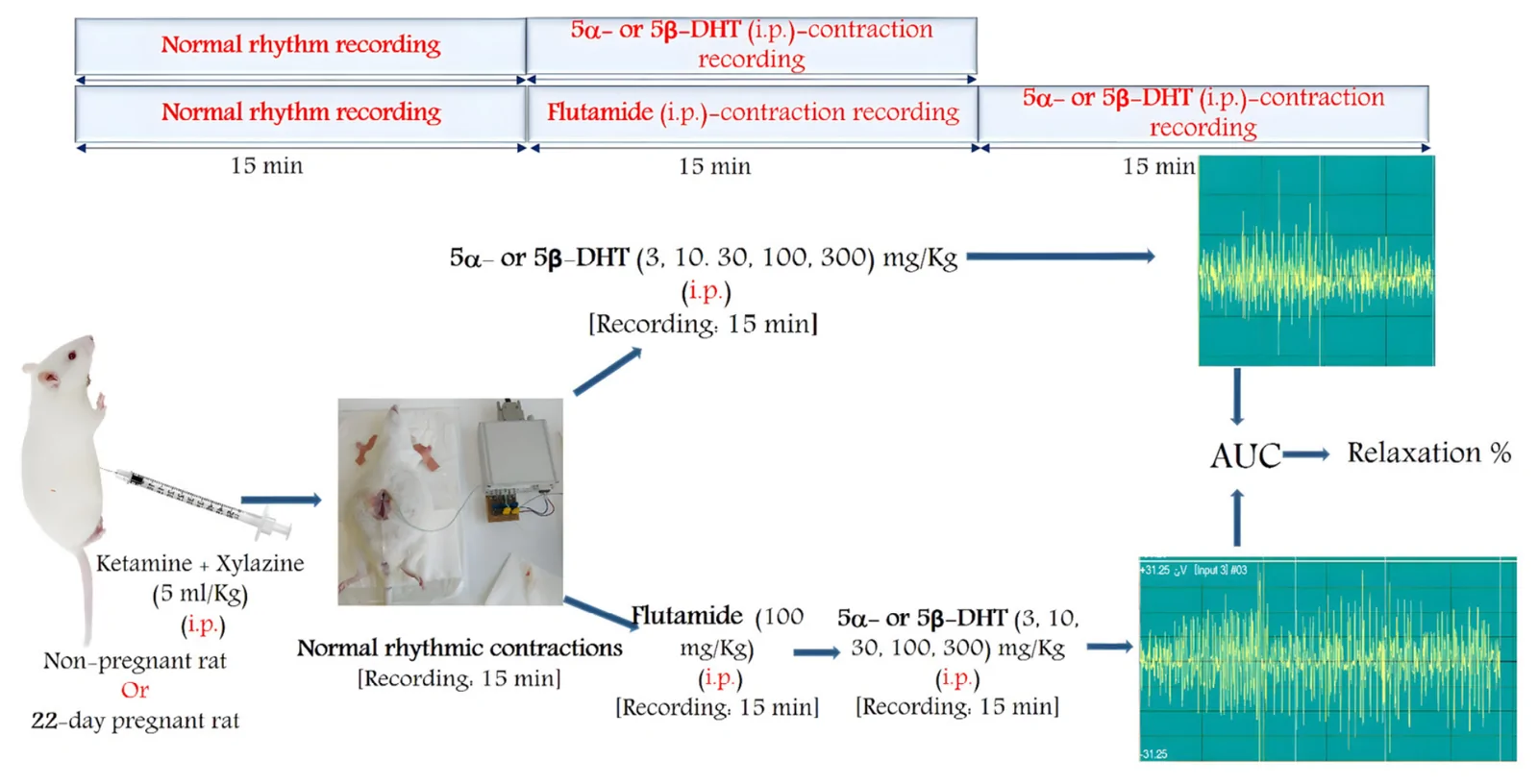
The schematic diagram of the in vivo investigation of the uterine action of 5α- or 5β-DHT in non-pregnant and 22-day-pregnant anesthetized rats.

**Figure 5:**
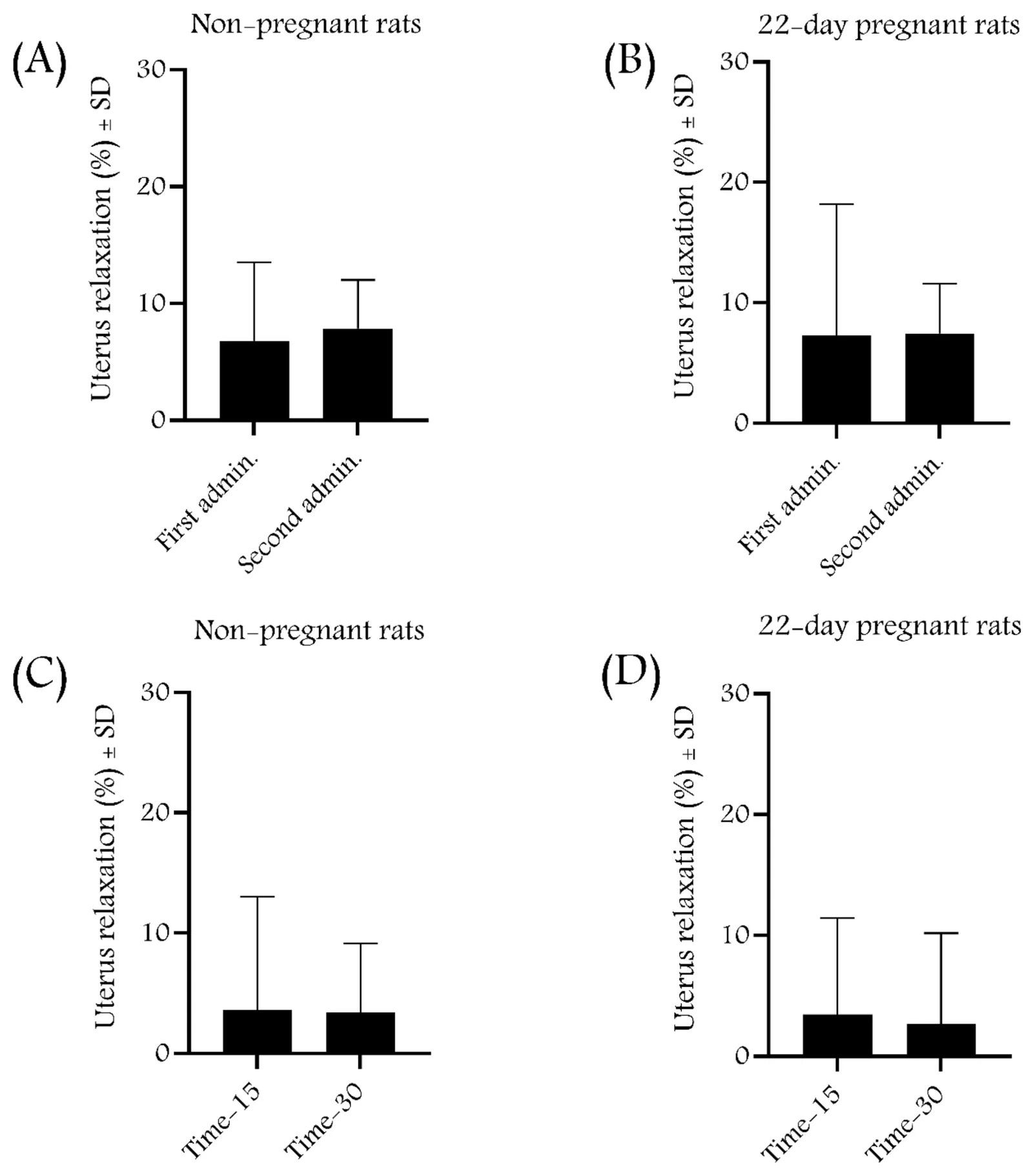
The in vivo effect of solvent and time passage on rat uterine muscle contraction. (**A**,**B**) are representative solvent effects; (**C**,**D**) represent the time passage effect in non-pregnant (**A**,**C**) and 22-day-pregnant rats (**B**,**D**), n = 5–6 per group. Data are expressed as the relaxation percentage mean ± SD; First admin., first administration of solvent; Second admin., second administration of solvent dose.

Non-pregnant and 22-day-pregnant rats were anesthetized using i.p.-administered ketamine + xylazine (36 + 4 mg/kg in 20 mL solution) in a volume of 5 mL/kg. After laparotomy, a strain gauge sensor (MSB-MET Ltd., Balatonfüred, Hungary) was placed on the uterine surface of the rat with sutures [30]. The rats were divided into 6 experimental groups: (1) solvent control; (2) 5α-DHT; (3) 5β-DHT; (4) 5α-DHT + flutamide; (5) 5β-DHT + flutamide; and (6) absolute control (n = 5–9/group). The animals received a single i.p. dose of 5α- or 5β-DHT alone (3, 10, 30, 100 or 300 mg/kg) or with flutamide (100 mg/kg). The solvent control group received DMSO + Macrogol 400 (25% + 75%) (1 mL/kg, i.p.); the absolute control group received physiological saline (1 mL/kg, i.p.). The contractions were measured and recorded at 15-minute intervals before and after dose administration using the S.P.E.L. Advanced IsoSys software (MSB-MET Ltd., Balatonfüred, Hungary), and the area under the curve (AUC) was calculated and expressed as response percentage. Based on the AUC changes measured for each dose, dose–response curves were plotted, and the doses causing 50% maximum effect (ED50) and maximum effect (Emax) were calculated. The solvent or the passage of time (fatigue test) alone did not cause any significant changes in contractions during the period investigated (Figure 3, Figure 4 and Figure 5). The rats were euthanized at the end of recording with a high dose of intravenous ketamine (150 mg/kg).

5α- or 5β-DHT were administered in different doses (3, 10, 30, 100 or 300 mg/kg i.p.) and with flutamide (100 mg/kg i.p.) using strain gauge sensors, the AUC was obtained, and the percentage of relaxation was calculated and analyzed.

### 2.10. Statistical Analysis

All data were analyzed using the Prism version 11.0 (GraphPad Software Inc., San Diego, CA, USA) computer program. Values were statistically evaluated with non-linear regression, an unpaired *t*-test (two-tailed) or a one-way ANOVA test (Dunnett’s post hoc test) with a significance level defined as *p* < 0.05. All data are expressed as mean ± SD.

## 3. Results

Details of the measurement data can be found in the Supplementary materials.

### 3.1. Results of the Isolated Organ Bath Studies

#### 3.1.1. The In Vitro Effect of 5α- and 5β-DHT on Uterine Contractions

**Figure 6:**
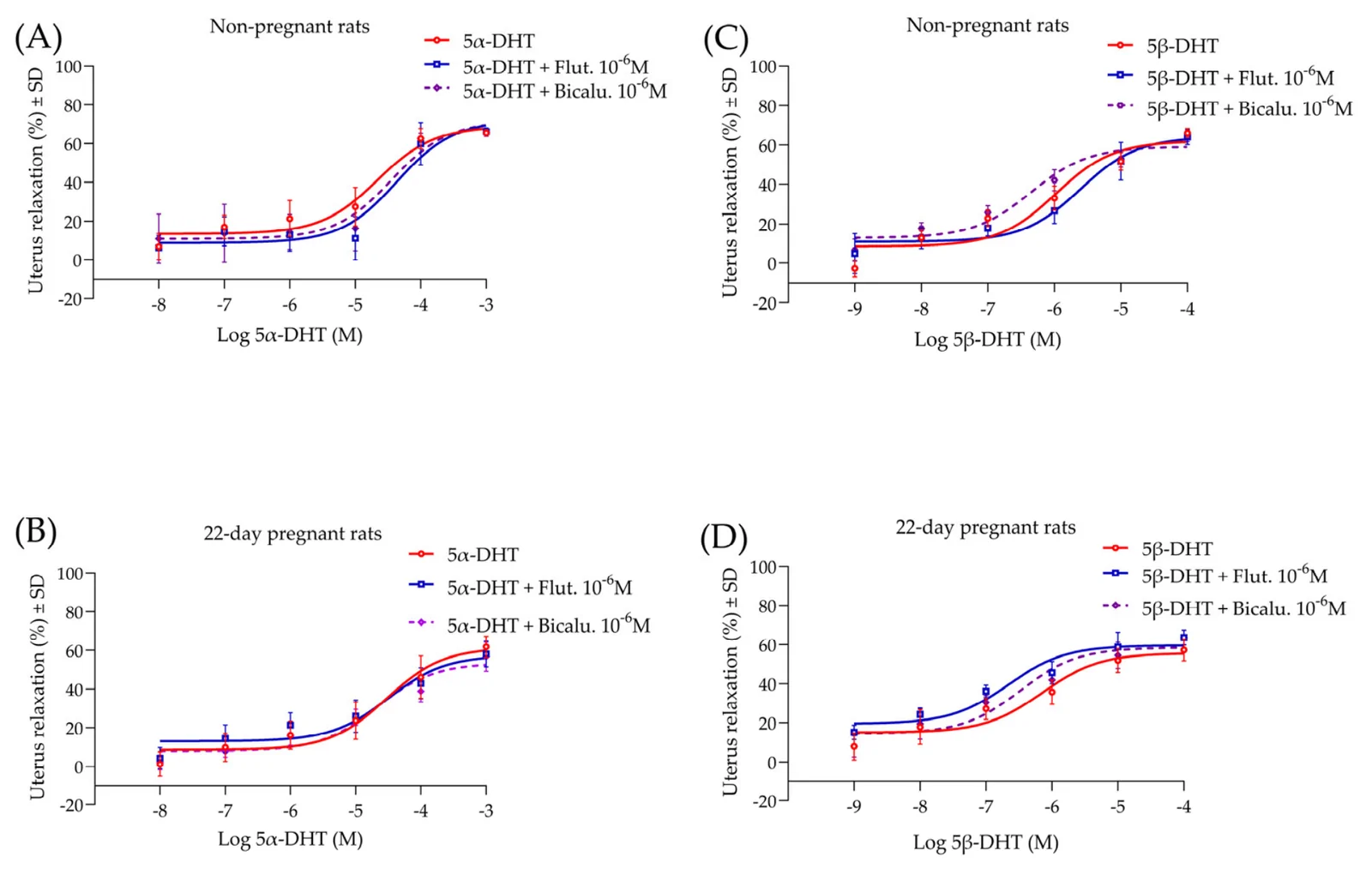
The in vitro effect of 5α-DHT (10^−8^–10^−3^ M) and 5β-DHT (10^−9^–10^−4^ M) on non-pregnant (**A**,**C**) and 22-day-pregnant rats (**B**,**D**). Uterine muscles were stimulated with KCl (25 mM) and pretreated with flutamide (10^−6^ M) or bicalutamide (10^−6^ M). Data are expressed as relaxation %. Bicalu., bicalutamide; DHT, dihydrotestosterone; Flut., flutamide.

**Figure 7:**
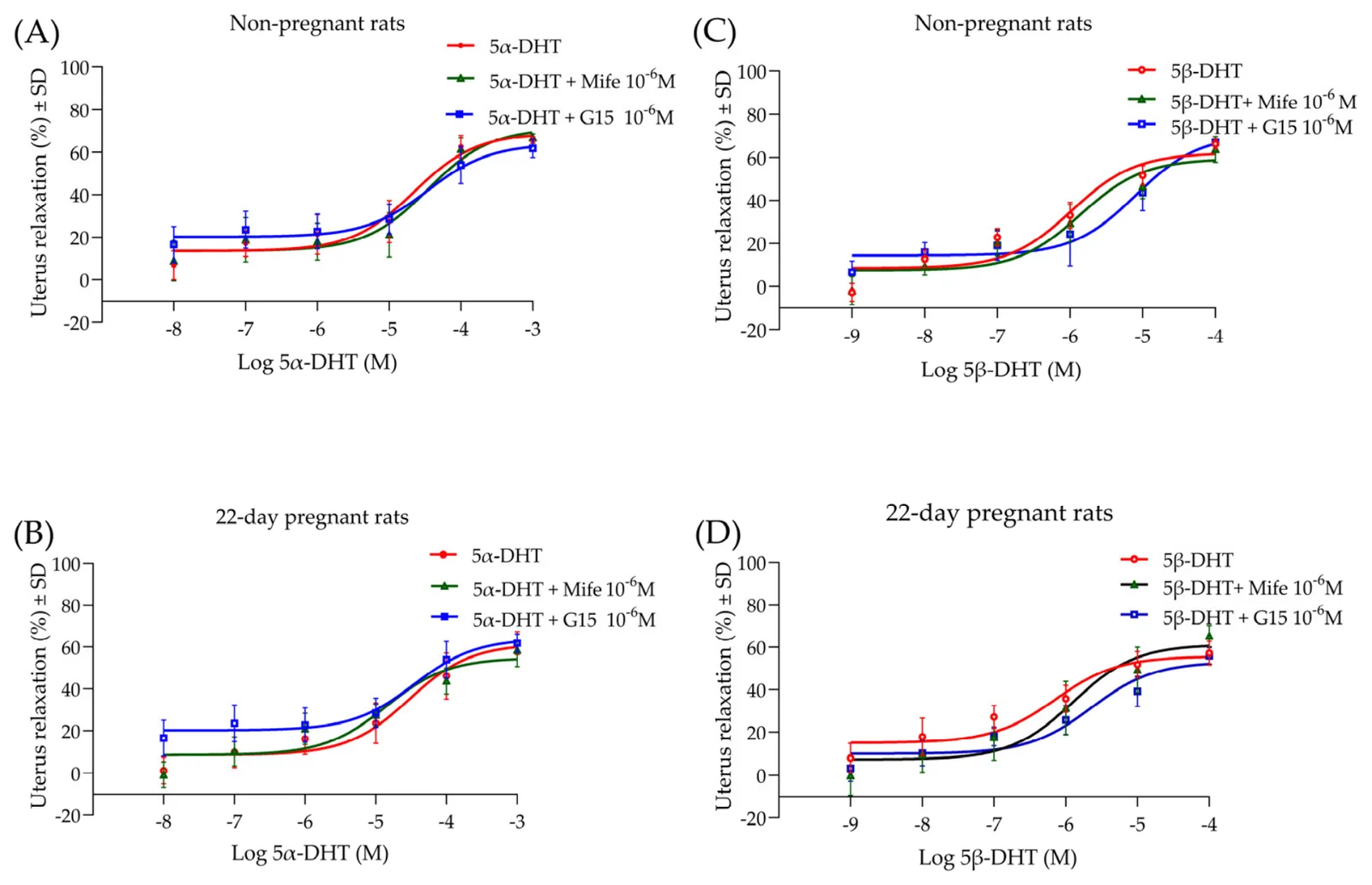
The in vitro effect of 5α-DHT (10^−8^–10^−3^ M) and 5β-DHT (10^−9^–10^−4^ M) on non-pregnant (**A**,**C**) and 22-day-pregnant rats (**B**,**D**). Uterine muscles were stimulated with KCl (25 mM) and pretreated with mifepristone (10^−6^ M) or G15 (10^−6^ M). Data are expressed as relaxation %. DHT, dihydrotestosterone; Mife, Mifepristone.

**Figure 8:**
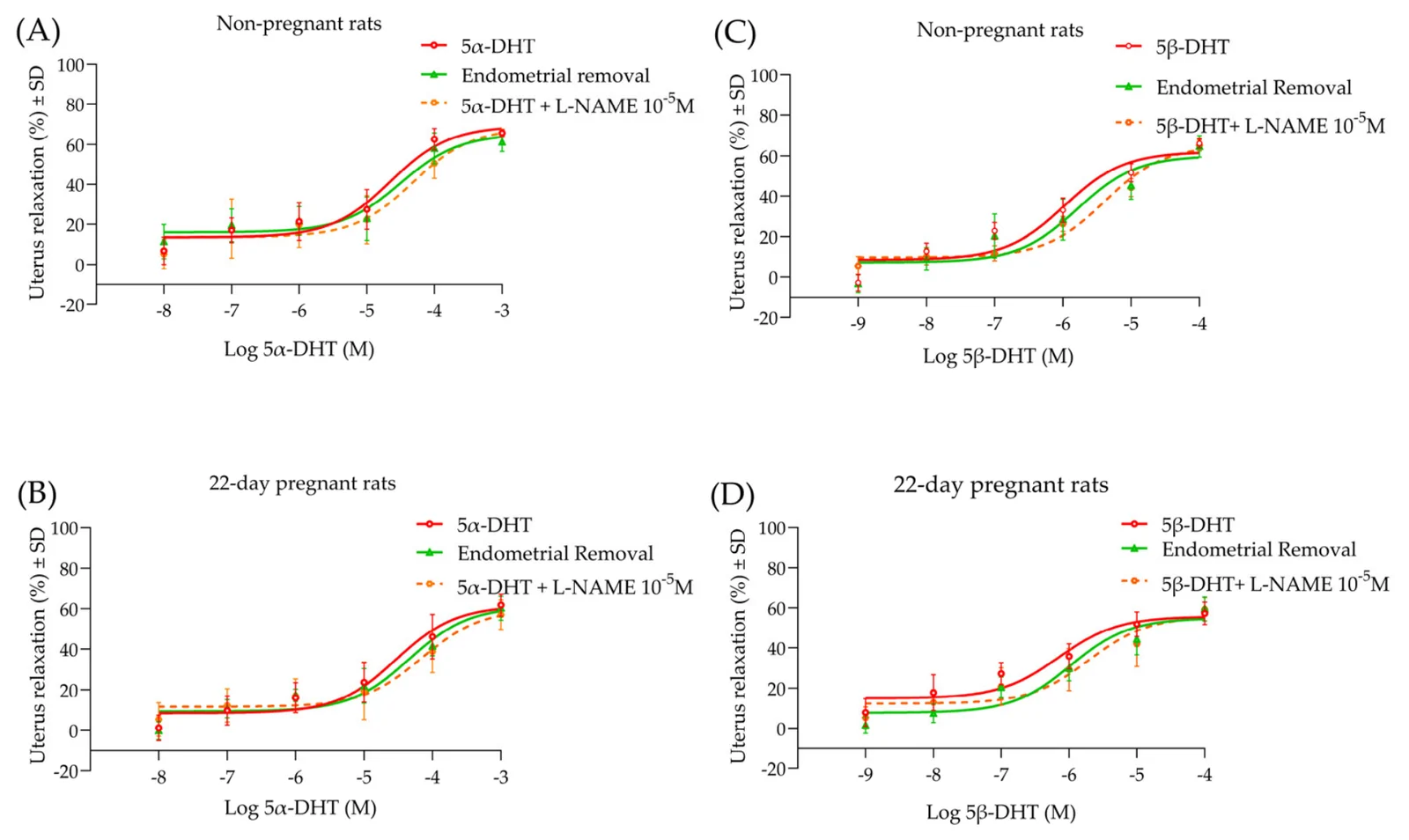
The in vitro effect of 5α-DHT (10^−8^–10^−3^ M) and 5β-DHT (10^−9^–10^−4^ M) on non-pregnant (**A**,**C**) and 22-day-pregnant rats (**B**,**D**). Uterine muscles were stimulated with KCl (25 mM) and pretreated with L-NAME (10^−5^ M), or after endometrial removal. Data are expressed as relaxation %. DHT, dihydrotestosterone; L-NAME, Nω-nitro-L-arginine methyl ester.

**Figure 9:**
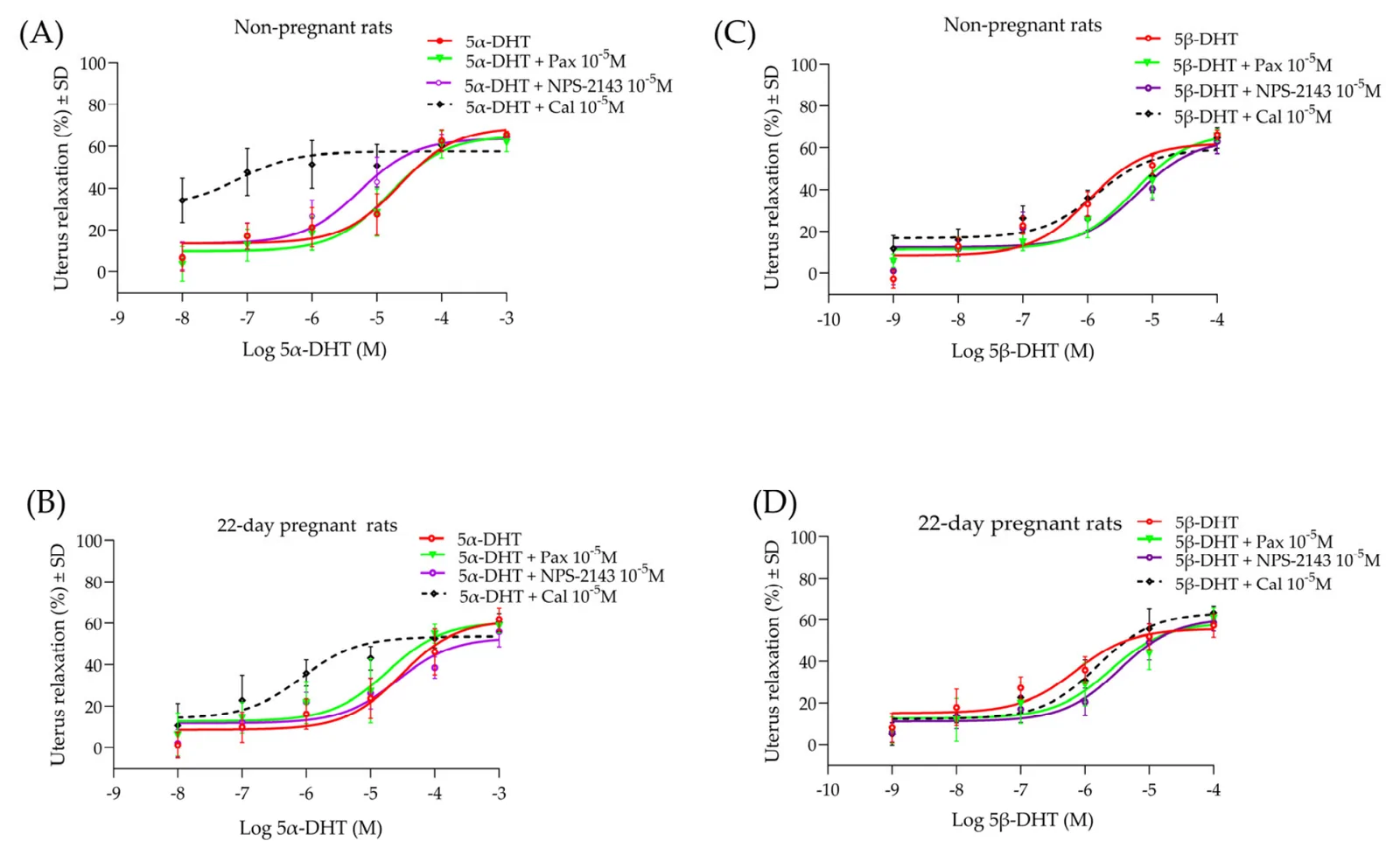
The in vitro effect of 5α-DHT (10^−8^–10^−3^ M) and 5β-DHT (10^−9^–10^−4^ M) on non-pregnant (**A**,**C**) and 22-day-pregnant rats (**B**,**D**). Uterine muscles were stimulated with KCl (25 mM) and pretreated with paxilline (10^−5^ M), NPS-2143 (10^−5^ M), or calindol (10^−5^ M). Data are expressed as relaxation %. Cal, calindol; DHT, dihydrotestosterone; Pax, paxilline.

5α- and 5β-dihydrotestosterone (5α- and 5β-DHT) showed an in vitro concentration-dependent inhibition of uterine muscle contractions induced by KCl (25 mM). The maximum relaxant effect of 5α-DHT is higher than that of 5β-DHT in non-pregnant and pregnant rats, with 5β-DHT showing lower EC_50_ values than 5α-DHT. The relaxing effect was lower in the pregnant compared to the non-pregnant rats in both DHTs. Flutamide, bicalutamide, mifepristone, paxilline, NPS-2143-HCl and the removal of the endometrium did not affect the relaxation effects of DHTs. G15 produced a right shift without affecting the E_max_ on the 5α-DHT curves in 22-day-pregnant rats, while N(ω)-nitro-L-arginine methyl ester (L-NAME) shifted both 5α- and 5β-DHT curves to the right in non-pregnant and 22-day-pregnant rats. However, the presence of calindol reduced the EC_50_ of 5α-DHT in non-pregnant and 22-day-pregnant rats and reduced its E_max_ in non-pregnant rats (Figure 6, Figure 7, Figure 8 and Figure 9, Table 1).

#### 3.1.2. 5α- and 5β-DHT Block CaCl_2_ Effect on Uterine Smooth Muscles

**Figure 10:**
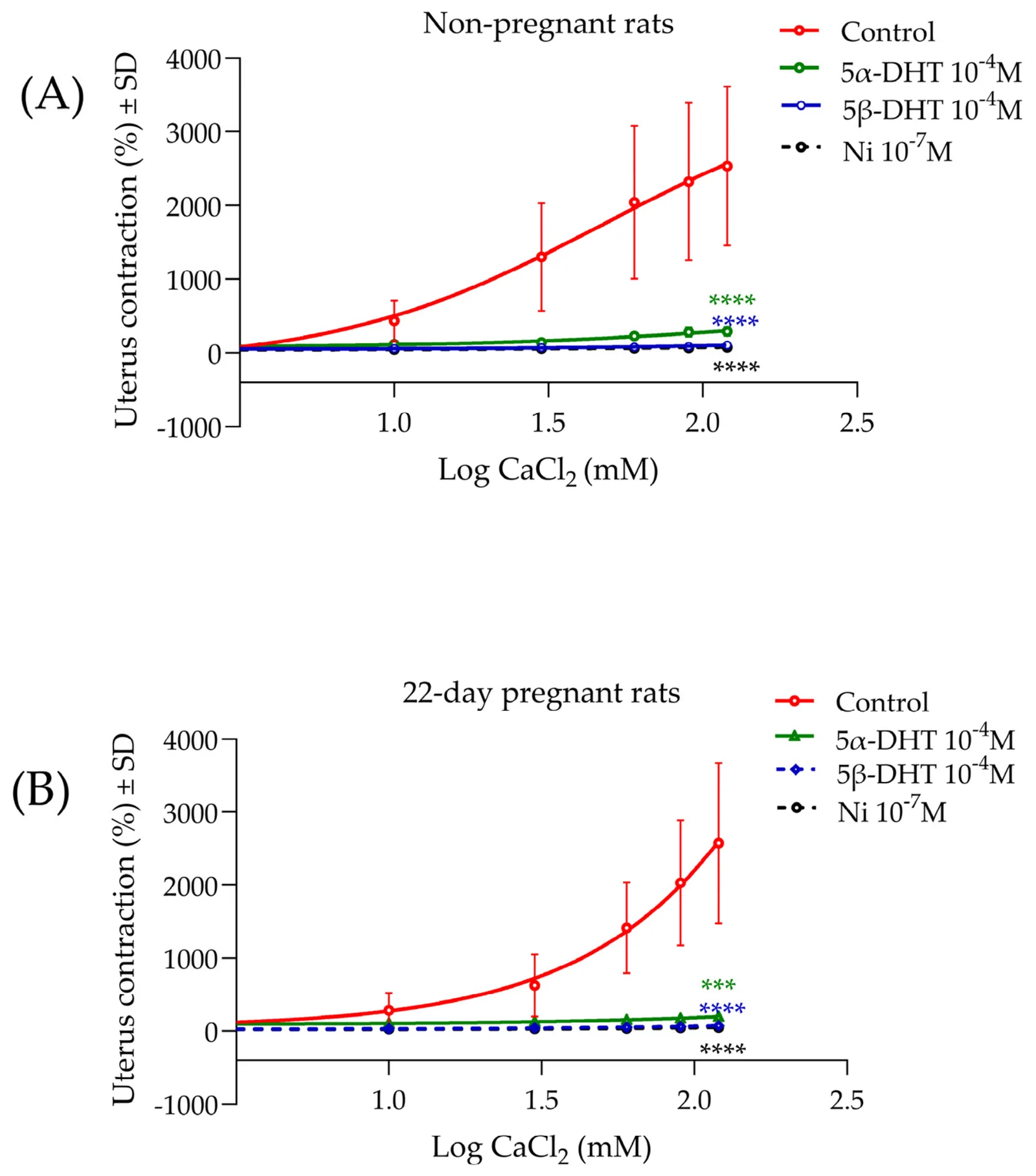
The inhibitory effect of 5α- and 5β-DHT and nifedipine on non-pregnant (**A**) and 22-day-pregnant uterine (**B**) tissues in vitro (n = 4–8). The contractions were initiated with KCl (40 mM) in a Ca^2+^-free buffer. Cumulative administration of CaCl_2_ initiated uterine contractions, which were completely blocked by 5α-, 5β-DHT and nifedipine. ***: *p* < 0.001, **** *p* < 0.0001 compared to the CaCl_2_ curve. Data were analyzed using one-way ANOVA test (Dunnett’s post hoc test) and are expressed as contraction % mean ± SD; CaCl_2_: calcium chloride; DHT, dihydrotestosterone; Ni, nifedipine.

Cumulative administration of calcium chloride (CaCl_2_) provoked and increased contractions stimulated by KCl (40 mM). A concentration of 5α- and 5β-DHT (10^−4^ M) or nifedipine (10^−7^ M) inhibited these contractions in non-pregnant and 22-day-pregnant rats (Figure 10).

### 3.2. cAMP Study

**Figure 11:**
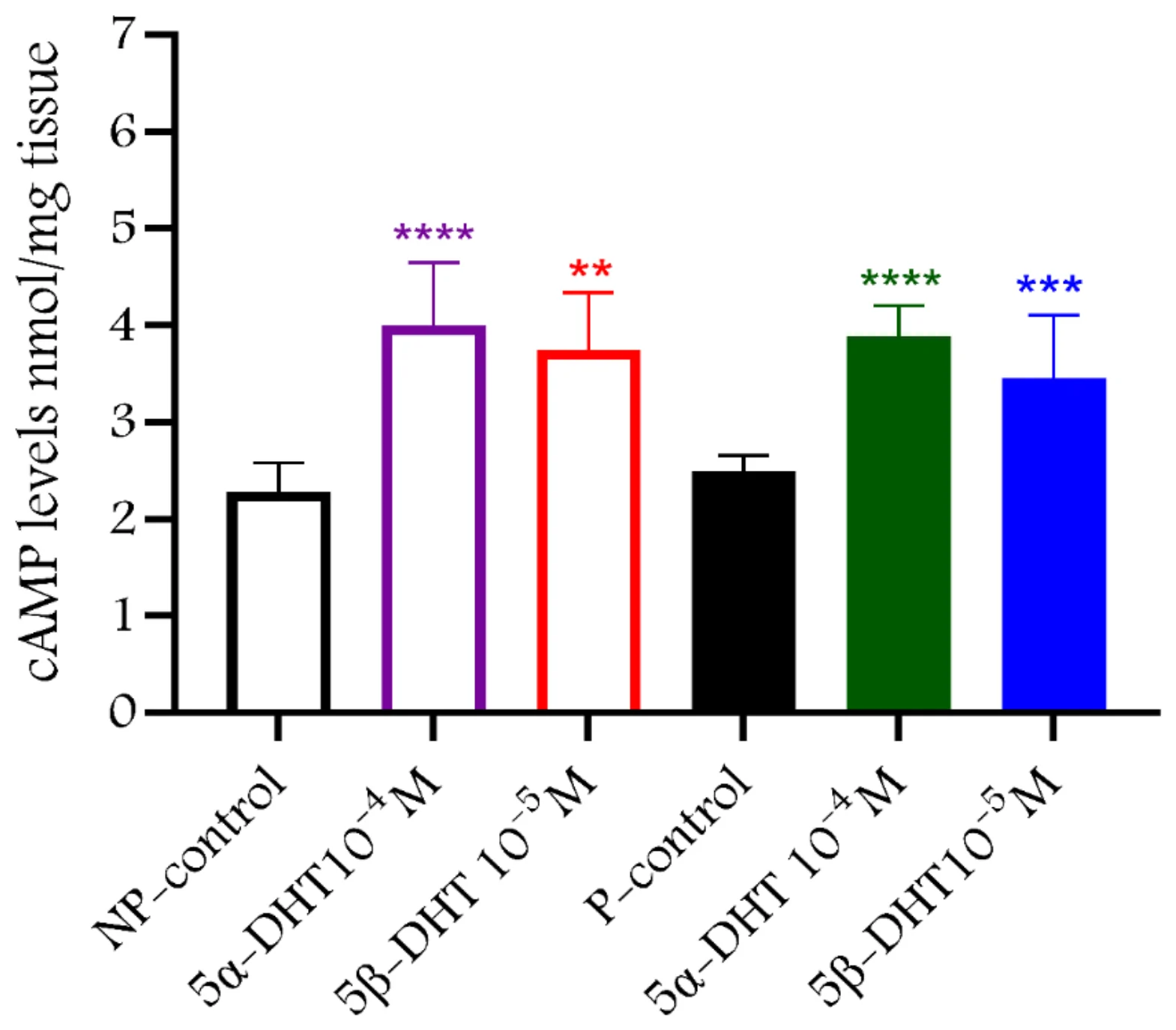
Change in uterine cAMP levels in the presence of 5α-DHT (10^−4^ M) or 5β-DHT (10^−5^ M) in non-pregnant (empty columns) and 22-day-pregnant (filled columns) rats (n = 6–9/group). The cAMP levels are expressed in nmol/mg tissue; DHT, dihydrotestosterone; NP, non-pregnant; P, 22-day-pregnant rats. **, *p* < 0.01; ***, *p* < 0.001; ****, *p* < 0.0001; compared to the corresponding control mean. Data were analyzed using one-way ANOVA test (Dunnett’s post hoc test).

The single dose of 5α-DHT (10^−4^ M) and 5β-DHT (10^−5^ M) increased cyclic adenosine monophosphate (cAMP) levels of uterine tissues in non-pregnant and 22-day-pregnant rats compared to control levels (n = 6–9 per group). No difference was found between 5α- and 5β-DHT actions (Figure 11).

### 3.3. cGMP Study

**Figure 12:**
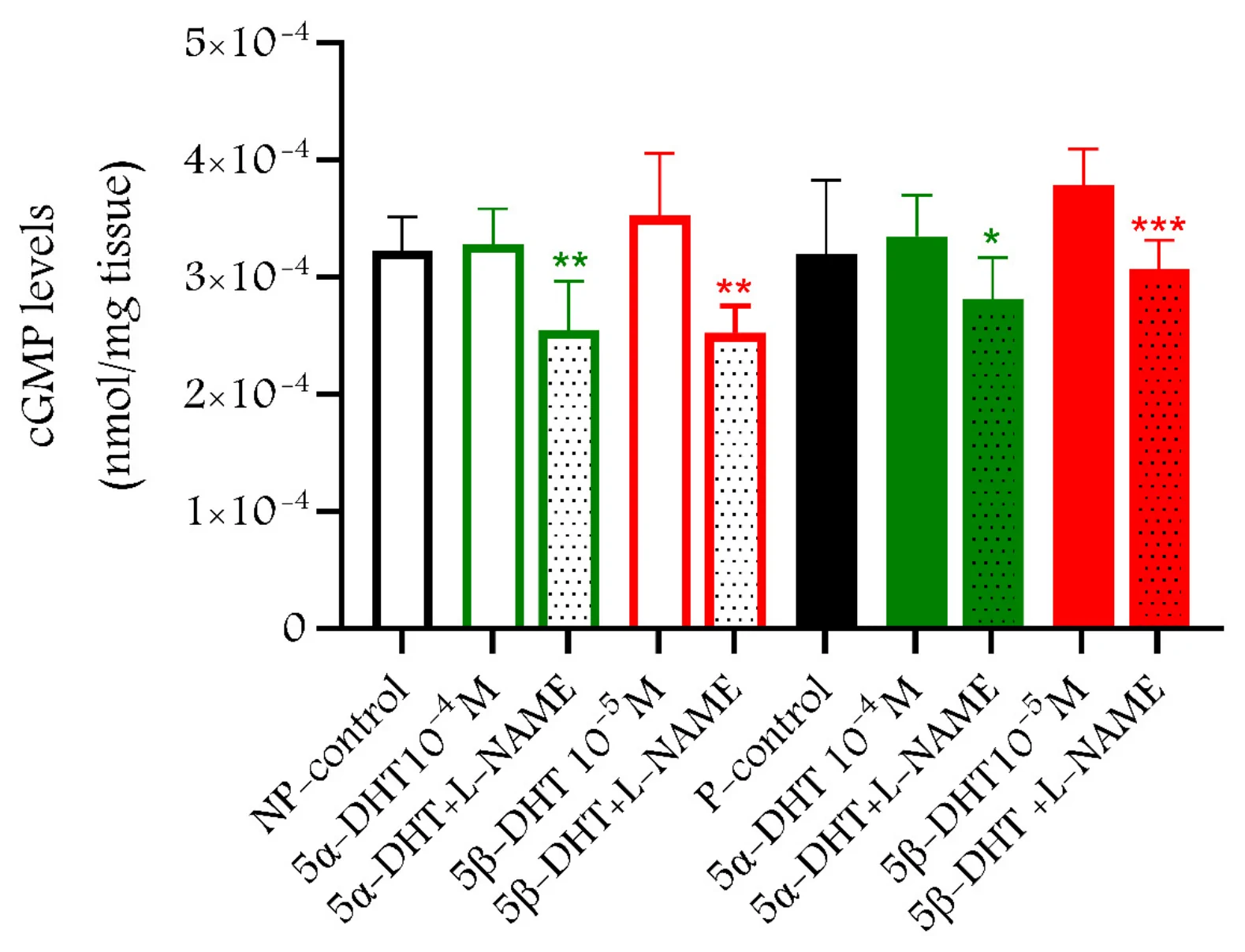
The uterine cGMP levels in the presence of 5α-DHT (10^−4^ M) or 5β-DHT (10^−5^ M) with or without L-NAME (10^−5^ M) in non-pregnant (empty columns) and 22-day-pregnant (filled columns) rats (n = 6–9/group). The cGMP levels are expressed in nmol/mg tissue; DHT, dihydrotestosterone; L-NAME, N(ω)-nitro-L-arginine methyl ester; NP, non-pregnant; P, 22-day-pregnant rats. Data were analyzed using one-way ANOVA test (Dunnett’s post hoc test) compared to the corresponding control *, *p* < 0.05, **, *p* < 0.01; ***, *p* < 0.001.

The levels of cyclic guanosine monophosphate (cGMP) in the non-pregnant and 22-day-pregnant uteri did not change in the presence of 5α-DHT (10^−4^ M) and 5β-DHT (10^−5^ M) compared to the control levels (n = 6–9 per group). No difference in cGMP levels was found between 5α-DHT and 5β-DHT. The presence of L-NAME reduced the increasing effects of DHTs on cGMP in both non-pregnant and 22-day-pregnant uteri (Figure 12).

### 3.4. 5α-and 5β-DHT Pharmacokinetics in Female Rats

**Figure 13:**
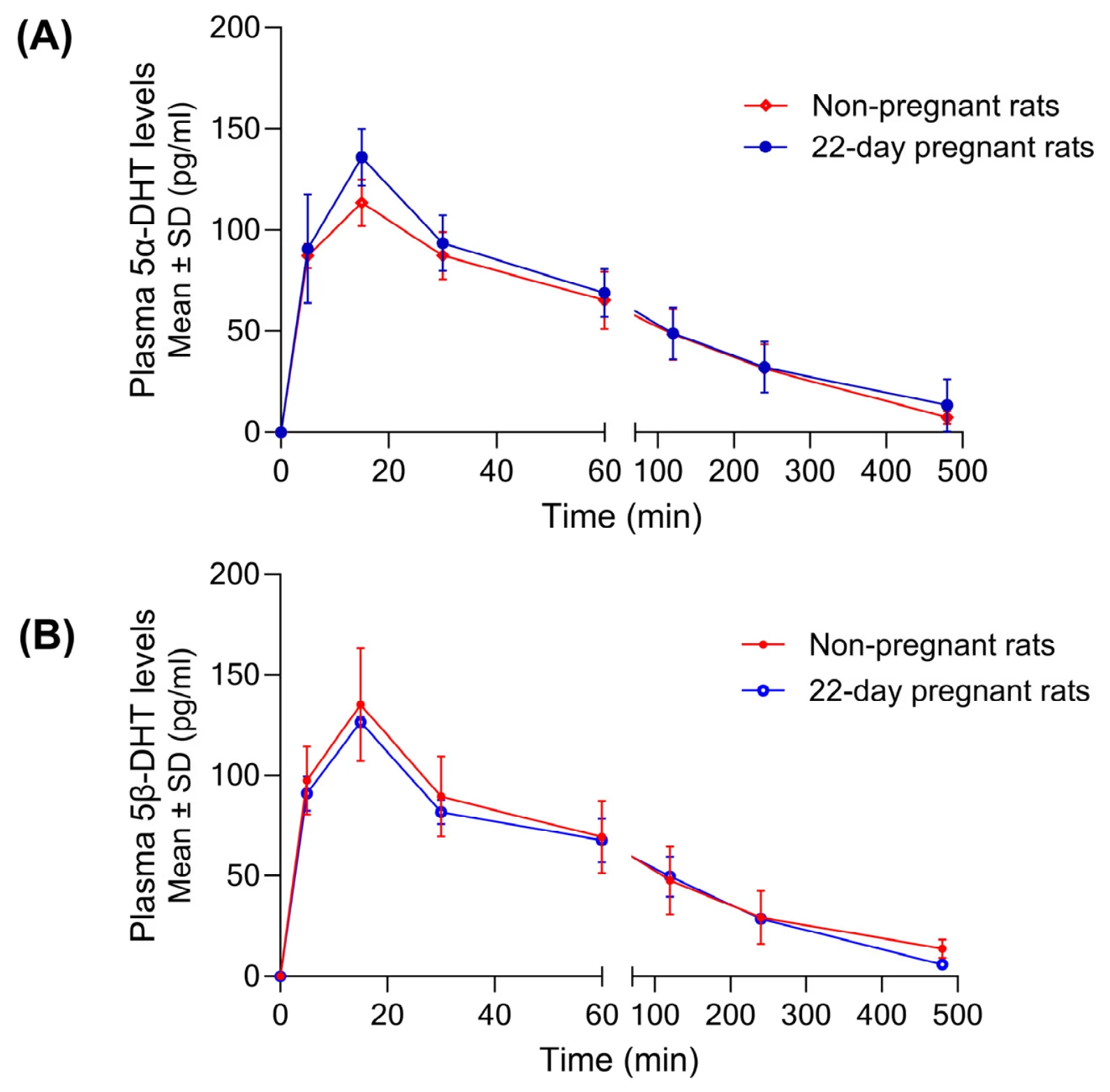
Plasma concentration–time curves of (**A**) 5α-DHT and (**B**) 5β-DHT after a single i.p. injection (10 mg/kg) in non-pregnant and 22-day-pregnant rats (n = 4–5 per group). DHT levels are expressed as pg/mL.

A single intraperitoneal (i.p.) dose of 5α- or 5β-DHT (10 mg/kg) resulted in a rapid increase in plasma DHT levels followed by a gradual decline in both non-pregnant and 22-day-pregnant rats. Maximum concentrations were achieved at 15 min in both groups, with higher peak values in 22-day-pregnant rats for 5α-DHT. In the case of 5β-DHT, the half-life was shorter in pregnant rats than in non-pregnant rats, while in non-pregnant rats it was longer than that of 5α-DHT. There was no difference in plasma levels of 5α-DHT and 5β-DHT in either non-pregnant or pregnant rats (Figure 13, Table 2).

**Figure 14:**
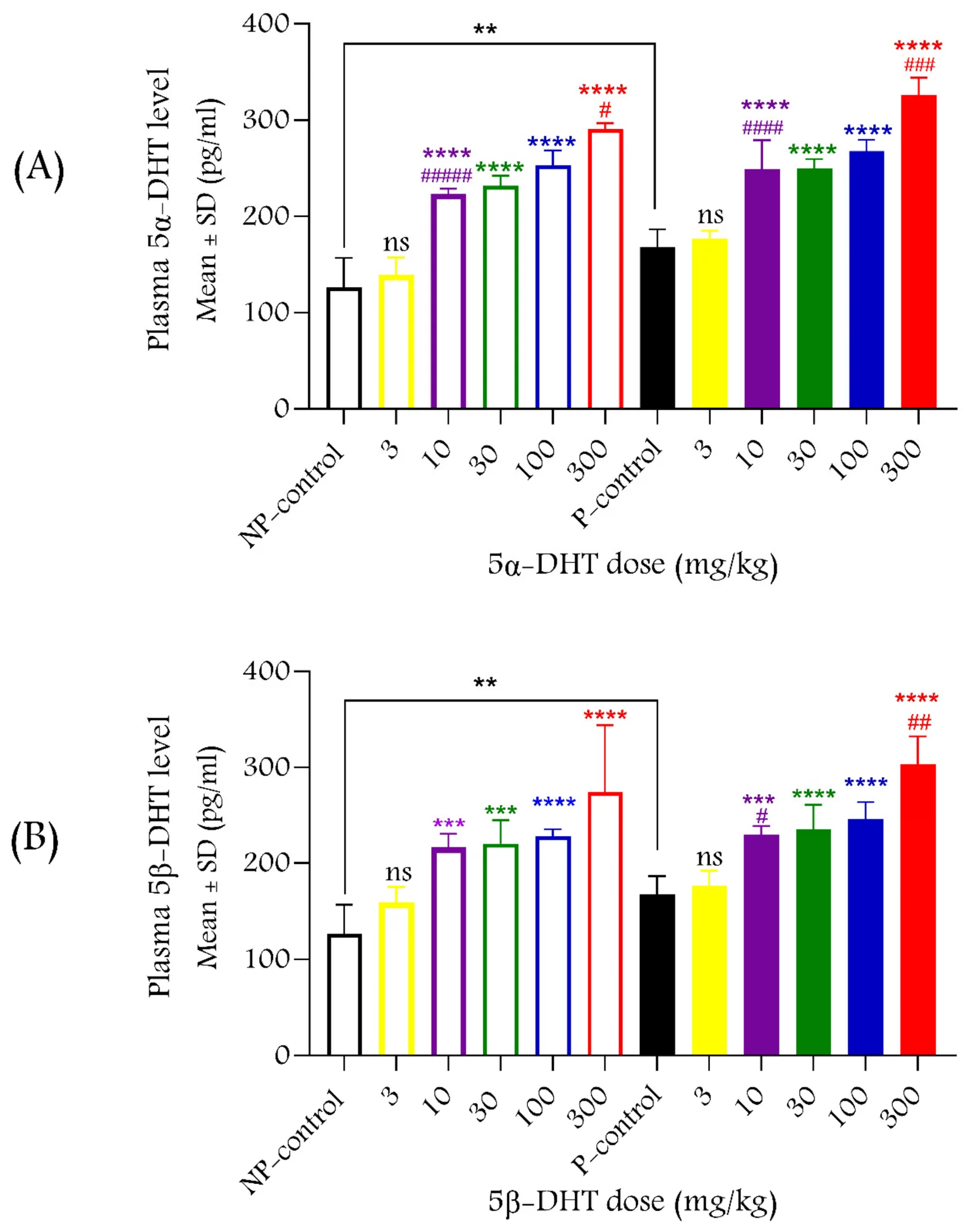
Plasma 5α- and 5β-DHT levels before and 30 min after the single i.p. dose in non-pregnant and 22-day-pregnant rats (n = 5–6 per group). The data were analyzed using ANOVA–Tukey’s multiple comparisons test compared to the control values within the group. Plasma levels are expressed as pg/mL; data are expressed as mean ± SD. **: *p* < 0.01, ***: *p* < 0.001, **** *p* < 0.0001 compared to the corresponding control. Data were analyzed using one-way ANOVA test (Dunnett’s post hoc test). ^#^: *p* < 0.05, ^##^: *p* < 0.01, ^###^: *p* < 0.001, ^####^ *p* < 0.0001 compared to the previous direct dose value. Data were analyzed using an unpaired t-test. The empty columns represent the non-pregnant rats; the filled columns represent the 22-day-pregnant groups. DHT: dihydro-testosterone; NP: non-pregnant; P: 22-day-pregnant; ns: non-significant compared to the corresponding controls.

Increasing the dose of the compounds raised plasma 5α- or 5β-DHT levels in non-pregnant and pregnant rats 30 min after intraperitoneal administration, although not all doses caused a significant increase in plasma levels compared to the previous dose (Figure 14).

### 3.5. Uterine Relaxing Effect of 5α- and 5β-DHT In Vivo

**Figure 15:**
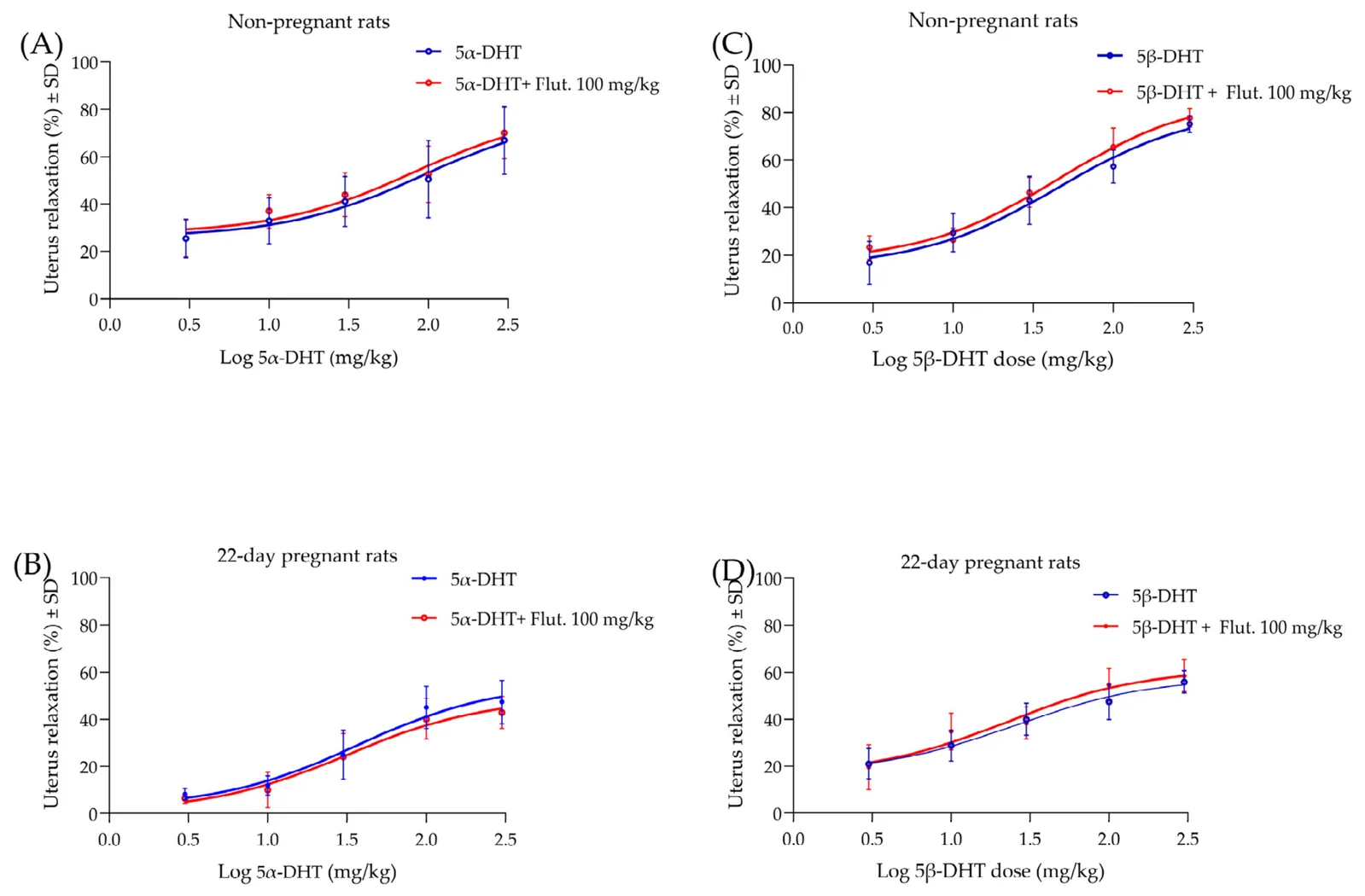
The in vivo non-genomic uterine-relaxing effect of 5α-DHT (**A**,**B**) and 5 β -DHT (**C**,**D**) in non-pregnant and late-pregnant rats (n = 8–9 per group). The single i.p. dose of 5α-or 5β-DHT induced a dose-dependent but flutamide-resistant uterine-relaxing effect in both non-pregnant (**A**,**C**) and late-pregnant (**B**,**D**) rats. Each point is the result of a single 5α-, 5β-DHT or + flutamide. DHT, dihydrotestosterone; Flut., flutamide.

The single administration of 5α- and 5β-DHT (3, 10, 30, 100 or 300 mg/kg i.p.) elicited an in vivo uterine-relaxing effect that is dose-dependent in both non-pregnant and 22-day-pregnant rats. This effect remained unchanged in the presence of the androgenic antagonist flutamide (100 mg/kg, i.p.). In our study, 5β-DHT showed significantly higher efficacy and potency compared to 5α-DHT in 22-day-pregnant rats. In addition, both DHTs showed a higher relaxation effect in non-pregnant rats than in pregnant rats (Figure 15, Table 3).

## 4. Discussion

The effects of sex hormones on numerous tissues have been studied extensively in the literature. In addition to their nuclear (genomic) action, they have been shown to relax smooth muscle through a non-nuclear (non-genomic) pathway [10,30,31,32,33,34]. In vitro studies on rat uterine muscles showed that T has a dose-dependent non-genomic muscle-relaxant effect. Additionally, testosterone (T) has been shown to have a non-genomic relaxant action in human coronary arteries [35], umbilical arteries [30], peripheral vasculature and smooth muscle of the airway [10,12]. Moreover, the T metabolite 5α-dihydrotestosterone (5α-DHT) showed relaxant activity on guinea pig gallbladder strips [19]; likewise, the other T metabolite 5β-dihydrotestosterone (5β-DHT) showed a non-genomic relaxant effect on human umbilical arteries [18]. Our previously published work proved a dose-dependent relaxing effect of T on non-pregnant and 22-day-pregnant rat uteri [3,28].

5α- and 5β-DHT are active metabolites of the uterine relaxant T [11,14]; however, to date, no studies have been conducted on their non-genomic effects on uterine muscle in non-pregnant and 22-day-pregnant rats. Consequently, the main objective of this study was to investigate the rapid, non-genomic effects of these T metabolites on uterine contractility under both in vitro and in vivo conditions. It has been well-established that a 30 min exposure duration is insufficient to induce genomic responses; therefore, all experiments were limited to a maximum of 30 min [3,10,36].

Both 5α- and 5β-DHT induced a dose-dependent inhibitory effect of KCl-induced contractions on non-pregnant and pregnant uterine muscles; 5α-DHT had a higher maximum effect, while 5β-DHT had better affinity (lower EC_50_ value), which is consistent with previous findings regarding smooth muscle [11,18,37].

The relaxing effects of 5α- or 5β-DHT were not blocked by the androgen receptor (AR) antagonist flutamide, suggesting that their actions are independent of AR. This is consistent with previously established T studies [3,28]. This, along with their rapid action (less than 30 min), confirms their non-genomic pathway [10]; therefore, the AR is essential in the genomic effects of androgens [9].

The non-genomic action of androgens might arise through many targets, including zinc transporter 9 (ZIP9), membrane progesterone receptor (mPR), G-protein estrogen receptor (GPER), large conductance, calcium-activated potassium channel (BKCa^2+^), voltage-gated calcium channel (VGCC), calcium-sensing receptor (CaSR) and nitric oxide synthase (NOS) [10]. Therefore, we investigated the effect of DHTs in the presence of blockers or modulators of these targets. The relaxing effects of 5α- or 5β-DHT were not inhibited by the selective ZIP9 inhibitor bicalutamide (also an AR blocker), the mPR antagonist mifepristone (also a glucocorticoid receptor blocker), and the selective BKCa^2+^ blocker paxilline; thus, these mechanisms do not seem to play a role in the non-genomic effects of DHTs in the uterus. The removal of the endometrium was also ineffective, so the endometrium also seems neutral in the non-genomic actions of these T metabolites.

The GPER inhibitor G15 produced a right shift only in the 5α-DHT concentration–response curve in the 22-day-pregnant rats, suggesting that GPER plays a role only in the uterine-relaxing effect of 5α-DHT. This stereoisomer-specific activation of GPER is already known, as 17β-estradiol activates the receptor, whereas 17α-estradiol does not [38]. However, our study is the first to describe this phenomenon in relation to the stereoisomers of DHT.

Although the CaSR antagonist NPS-2143 did not modify the actions of DHTs, the presence of CaSR activator calindol improved the affinity of 5α-DHT in non-pregnant and 22-day-pregnant rats, while slightly reducing the E_max_ in non-pregnant animals. Since CaSR activation couples G-protein signaling that modulates the activity of voltage-operated calcium channels (VOCCs) and intracellular calcium, 5α-DHT seems to influence CaSR-associated regulation. This suggests a possible receptor-independent pathway in the mechanism of action of 5α-DHT, which reduces the open state or conductance of VOCCs [39,40,41]. It is not clear why the antagonist did not alter the action of 5α-DHT. NPS-2143 has been found to directly inhibit VOCCs in the smooth muscle of the gastrointestinal tract [42], so we assume that this action is overwhelmed by the Ca^2+^ blocking effect of 5α-DHT in the uterus.

T has previously demonstrated to inhibit Ca^2+^ channels [11,18,28], and it has been found that 5β-DHT also induces vasodilation in a similar manner [37]. The single addition of 5α- or 5β-DHT blocked the contractions induced by a gradual increase in calcium chloride (CaCl_2_) concentrations, starting from a Ca^2+^-free environment. The degree of blockade was comparable to that of nifedipine, suggesting that DHTs are Ca^2+^ channel blockers, and this may be one of the main components of their non-genomic uterine-relaxant effect. This result supports previous findings that DHT stereoisomers inhibit Ca^2+^ influx [43,44], but our result provides the first evidence of this in uterine smooth muscle.

Both 5α- and 5β-DHT increased cAMP levels in non-pregnant and 22-day-pregnant uteri, confirming that stimulation of G_s_ via G-protein-coupled receptors plays an important role in their effects [3,45]. In the case of c, stimulation of the GPER receptor may be responsible for the increase in cAMP levels and uterine relaxation in pregnant rats; however, we were unable to identify such a receptor mechanism in non-pregnant rats in response to 5α-DHT or in either pregnant or non-pregnant rats in response to 5β-DHT. The effect of 5β-DHT in increasing cAMP levels is a true novelty, as the compound was previously believed to exert its effects exclusively through Ca^2+^ channels [44]. A common target for both 5α- and 5β-DHT could be the membrane-associated progesterone receptor family [46]; however, there is currently no evidence to support this hypothesis.

In the presence of DHTs, the levels of cGMP remained unchanged; however, the addition of the NOS inhibitor L-NAME reduced the levels of cGMP in the uteri and enhanced the relaxing EC50 values of the compounds. Additionally, endometrial removal did not modify the relaxing effects of DHTs, making it difficult to describe the clear role of NOS and nitric oxide in the non-genomic mechanisms of action of DHTs, as the endometrium is the main site for endothelial NOS (eNOS) activity in the uterus, but the myometrium is also capable of producing it [47]. It is highly likely that NOS inhibition plays a partial role in the uterine-relaxing mechanisms of DHTs; the presence of L-NAME alone reduces cGMP levels and improves contractions, thus reducing the efficacy of these compounds. The calcium-blocking effect—along with the cAMP and GPER findings—and the NOS-inhibiting effect, together with the results of the previously mentioned studies [11,18,28,37], extend our observations to uterine muscle and suggest that the non-genomic effect involves more complex mechanisms. The involvement of cAMP, GPER, and NOS in the myometrium suggests that the uterine muscle possesses additional regulatory pathways that contribute to the rapid relaxing effect. Some of these mechanisms have been identified individually in vascular, airway, gastrointestinal, gallbladder and genital smooth muscle [48,49,50,51,52], but they have not yet been identified collectively in any other type of smooth muscle. In this regard, the uterus appears to be unique.

We also investigated the uterine-relaxing effect of DHTs under in vivo conditions: we measured mechanical contractions in anesthetized, non-pregnant, and pregnant rats using a strain gauge placed on the uterus. The solvent for the DHTs was not suitable for intravenous administration, so intraperitoneal administration was chosen, which ensures rapid absorption and a rapid onset of action. Since no information was available on the basic pharmacokinetic parameters of 5α- and 5β-DHT in female rats, we conducted this study to ensure that the intended short-term, 30 min in vivo effect could be achieved after a single intraperitoneal injection. In both non-pregnant and pregnant rats, the injection of 5α- or 5β-DHT resulted in rapid elevation of plasma levels with appropriate elimination half-lives to maintain the effect for 30 min. Although slight differences in the parameters of the compounds were observed between pregnant and non-pregnant rats, it can generally be said that there is no significant difference in their pharmacokinetics.

Single doses of 5α- or 5β-DHT induced dose-dependent but androgen receptor antagonist-independent uterine relaxation in 30 min in non-pregnant and pregnant rats in vivo, which is consistent with our in vitro results and previous T-studies [3,11,28,53,54]. 5β-DHT exhibited lower ED_50_ values in both non-pregnant and pregnant rats, suggesting that it has a higher affinity for Ca^2+^ channels and G-protein-coupled receptor(s) than 5α-DHT. The maximum relaxant effects in vitro and in vivo were significantly lower in pregnant animals compared to non-pregnant rats for both DHTs. This might be explained by the higher expression of voltage-gated Ca^2+^ channels in the pregnant uterus [55]. Since, similar to their precursor T [3], one of the main mechanisms of DHTs to relax smooth muscle is to block these Ca^2+^ channels, a higher expression of these channels may contribute to a weaker effect of DHTs during pregnancy.

Plasma DHT levels were also measured 30 min after a single i.p. injection of different doses. Plasma levels of administered DHT significantly exceeded the control level, resulting in a 2- to 2.5-fold increase after the highest dose (300 mg/kg) and demonstrating that the observed relaxing effects were related to elevated DHT levels. Since DHTs are physiological hormone metabolites, it is possible that they might play a physiological role in the regulation of uterine contractions. However, such a role is unlikely, as the concentrations used in in vitro experiments (up to 10^−3^ M) and the doses used in in vivo experiments (up to 300 mg/kg) were pharmacological rather than physiological. The lowest effective dose (10 mg/kg in vivo) doubled, while the highest dose (300 mg/kg) nearly tripled the plasma levels of both DHT stereoisomers compared to physiological levels.

An important limiting factor in assessing the clinical relevance of our findings is the physiological difference between rat and human uterine muscle; although the rat uterus is a recognized model for studying uterine contractility, results obtained in rats cannot be directly extrapolated to humans. Differences in endocrine regulation observed during the late stages of pregnancy, as well as the expression and distribution of receptors and the expression and regulation of calcium channels, may influence the response of human uterine tissue to DHT. Therefore, the mechanisms identified in this study require further investigation using human uterine samples, which are essential for understanding the effects in humans.

Another limitation of our study is that we did not investigate the long-term action of 5α-DHT and 5β-DHT on uterine contractions and androgen-dependent proteins. Based on the high absorption rate and high lipid solubility of DHTs, they are expected to cross the placenta and the CNS, which could affect the fetus or mother, especially in multiple doses. Before considering DHTs for clinical use, it is crucial to conduct more studies on the harmful effects of DHTs on fetuses and mothers in addition to the CNS effects, with particular attention to neuroendocrine pathways.

## 5. Conclusions

5α-DHT and 5β-DHT exert a non-genomic inhibitory effect on uterine contractions, and their mechanisms of action are likely different. Both DHTs are likely to block Ca^2+^ channels, while 5α-DHT also acts via GPER in pregnant uterine tissue. This study provides new and initial evidence for the in vitro and in vivo uterine-relaxing effects of 5α-DHT and 5β-DHT during the late stages of pregnancy in rats. Their in vivo effects are proportional to their plasma levels, and their pharmacokinetic parameters (a relatively short half-life and the short time required to reach maximum plasma concentration) can provide a basis for further studies using human uterine tissue from pregnant women, aiming to determine their potential clinical application in conditions associated with uterine hyperactivity, confirm the identified mechanisms, and establish long-term safety for both mother and fetus. Since 5β-DHT is believed to have no genomic effects, its more water-soluble analogs would allow for more practical dosing regimens in clinical practice.

## Figures and Tables

**Table 1:** E_max_ and EC_50_ of 5α- and 5β-DHT in non-pregnant and 22-day-pregnant rats in vitro.

Parameters	Non-Pregnant Rats		22-Day-Pregnant Rats	
	E_max_	EC_50_	E_max_	EC_50_
*5α-DHT*	*69.1 ± 2.6*	*2.2 × 10^−005^ ± 2.3 × 10^−006^*	63.2 ± 9.9 **	*3.0 × 10^−005^ ± 7.6 × 10^−006^*
5α-DHT+Flut.	**73.8 ± 4.7 ^+^**	4.4 × 10^−005^ ± 1.1 × 10^−005^	58.8 ± 8.5	3.2 × 10^−005^ ± 2.2 × 10^−006^
5α-DHT+Bicalu.	71.7 ± 3.6	3.6 × 10^−005^ ± 2.2 × 10^−006^	57.2 ± 5.8	2.1 × 10^−005^ ± 7.6 × 10^−006^
5α-DHT+Mife	71.1 ± 3.4	3.5 × 10^−005^ ± 3.5 × 10^−006^	56.9 ± 8.6	1.8 × 10^−005^ ± 4.6 × 10^−006^
5α-DHT+G15	65.4 ± 5.3	3.5 × 10^−005^ ± 4.8 × 10^−006^	54.3 ± 4.6	**8.2 × 10^−005^ ± 3.0 × 10^−007 +^**
5α-DHT+End. rem.	65.1 ± 6.9	3.2 × 10^−005^ ± 5.4 × 10^−006^	63.4 ± 8.5	4.7 × 10^−005^ ± 8.8 × 10^−006^
5α-DHT+L-NAME	69.1 ± 4.2	**4.8 × 10^−005^ ± 8.3 × 10^−006 +^**	60.1 ± 6.1	**7.1 × 10^−005^ ± 3.6 × 10^−007 ++^**
5α-DHT+Pax	65.2 ± 4.1	2.0 × 10^−005^ ± 4.6 × 10^−006^	59.3 ± 6.6	1.8 × 10^−005^ ± 4.1 × 10^−006^
5α-DHT+NPS-2143	65.3 ± 3.1	5.4 × 10^−006^ ± 1.5 × 10^−006^	54.8 ± 8.1	2.6 × 10^−005^ ± 1.3 × 10^−006^
5α-DHT+Cal	**64.8 ± 3.1 ^++^**	**7.5 × 10^−008^ ± 7.6 × 10^−009 ++++^**	55.5 ± 3.8	**8.5 × 10^−007^ ± 6.5 × 10^−008 ++^**
	**E_max_**	**EC_50_**	**E_max_**	**EC_50_**
*5β-DHT*	***61.2 ± 6.2 ^####^***	***1.1 × 10^−006^ ± 3.8 × 10^−007 ####^***	56.0 ± 8.1 *^#^	***7.1 × 10^−007^ ± 4.8 × 10^−007 #^***
5β-DHT+Flut.	66.0 ± 4.4	2.4 × 10^−006^ ± 1.1 × 10^−007^	60.6 ± 6.1	2.2 × 10^−007^ ± 1.7 × 10^−007^
5β-DHT+Bicalu.	59.9 ± 3	4.4 × 10^−006^ ± 2.4 × 10^−008^	59.8 ± 5.5	3.3 × 10^−007^ ± 6.3 × 10^−008^
5β-DHT+Mife	59.7 ± 9.1	1.4 × 10^−006^ ± 6.4 × 10^−007^	62.4 ± 3.7	1.3 × 10^−006^ ± 6.0 × 10^−007^
5β-DHT+G15	69.8 ± 9.2	5.1 × 10^−006^ ± 3.4 × 10^−007^	55.3 ± 3.6	2.3 × 10^−006^ ± 1.7 × 10^−007^
5β-DHT+End. rem.	61.1 ± 9.6	1.6 × 10^−006^ ± 1.5 × 10^−007^	57.3 ± 8.1	1.1 × 10^−006^ ± 1.7 × 10^−007^
5β-DHT+L-NAME	65.9 ± 3.4	**4.1 × 10^−006^ ± 6.4 × 10^−007 +^**	58.8 ± 9.3	**2.0 × 10^−006^ ± 2.3 × 10^−007 +^**
5β-DHT+Pax	67.6 ± 6.7	5.3 × 10^−006^ ± 1.5 × 10^−007^	60.6 ± 9.8	2.4 × 10^−006^ ± 2.5 × 10^−007^
5β-DHT+NPS-2143	63.8 ± 9.6	5.9 × 10^−006^ ± 1.4 × 10^−007^	61.3 ± 6.1	3.4 × 10^−006^ ± 1.0 × 10^−006^
5β-DHT+Cal	64.9 ± 10.0	1.4 × 10^−006^ ± 4.1 × 10^−007^	62.6 ± 8.2	1.5 × 10^−006^ ± 3.5 × 10^−007^

**^+^**, *p* < 0.05, **^++^**, *p* < 0.01, **^++++^**, *p* < 0.0001 compared to the corresponding DHT values; Data were analyzed using one-way ANOVA test; *, *p* < 0.05; **, *p* < 0.01 compared to the corresponding non-pregnant values; ^#^, *p* < 0.05; ^####^, *p* < 0.0001, compared to the 5α-DHT values. Data were analyzed using an unpaired t-test. Bicalu., bicalutamide; Cal, calindol; DHT, dihydrotestosterone; End. rem., endometrial removal; EC_50_, concentration causing 50% maximum effect; E_max_, maximum effect; Flut., flutamide; L-NAME, N(ω)-nitro-L-arginine methyl ester; Mife, mifepristone; Pax, paxilline. Data are expressed as mean ± SD. Italic represents control values, while significant changes are highlighted in bold.

**Table 2:** Pharmacokinetic parameters of 5α- and 5β-DHT after a single 10 mg/kg i.p. injection in non-pregnant and pregnant rats.

Variable	Units	Non-Pregnant		22-Day-Pregnant Rats	
		Mean	±SD	Mean	±SD
	**5α-DHT**				
AUC	min*pg/mL	17,035.7	±4250.4	18,694.2	±6108.7
Clearance (Cl/F)	mL/min/kg	567.2	±137.6	504.7	±218.8
c_max_	pg/mL	113.6	±11.5	135.9 *	±14.1
t_1/2_	min	134.9	±24.1	182.6	±100.5
Elimination rate constant (λz)	1/min	0.005	±0.001	0.005	±0.003
t_max_	min	15	±0.0	15	±0.0
	**5β-DHT**				
AUC	min*pg/mL	18,383.0	±5798.3	16,679.2	±1832.8
Clearance (Cl)	mL/min/kg	475.2	±122.5	569.3	±67.2
c_max_	pg/mL	135.2	±28.1	126.6	±2.4
t_1/2_	min	**199.3 ^#^**	±47.6	120.8 **	±7.9
Elimination rate constant (λz)	1/min	0.004	±0.001	0.005	±0.0004
t_max_	min	15	±0.0	15	±0.0

*: *p* < 0.05, **: *p* < 0.01 compared to non-pregnant values. ^#^: *p* < 0.05 compared to the 5α-DHT values. AUC, area under the curve; C_max_, maximum concentration; t_1/2_, half-life; t_max_, time to maximum concentration; SD: standard deviation. Data were analyzed using an unpaired *t*-test. Significant changes are highlighted in bold.

**Table 3:** E_max_ and ED_50_ values of the uterine-relaxing dose–response curves of 5α- and 5β-DHT in non-pregnant and 22-day-pregnant rats (n = 8–9 per group). Data were analyzed using an unpaired t-test and are expressed as means ±SD.

Parameter	Non-Pregnant Rats		22-Day-Pregnant Rats	
	5α-DHT	5α-DHT + Flut.	5α-DHT	5α-DHT + Flut.
**E_max_ (%)**	78.3 ± 16.1	79.6 ± 16.8	55.3 ± 9 *	49.2 ± 6.8 *
**ED_50_(mg/kg)**	93.1 ± 4.3	81.8 ± 5.3	36.8 ± 3.2 ****	32.2 ± 2.02 ****
	**5β-DHT**	**5β-DHT + Flut.**	**5β-DHT**	**5β-DHT + Flut.**
**E_max_ (%)**	81.9 ± 22.8	87.1 ± 23.7	57.9 ± 14.0 *	61.7 ± 15.7 *
**ED_50_ (mg/kg)**	**45.0 ± 9.6 ^####^**	45.7 ± 9.1	25.8 ± 3.03 ***^####^	23.0 ± 1.8 ****^####^

*, *p* < 0.05, ****, *p* < 0.0001 compared to the corresponding non-pregnant values, ^####^, *p* < 0.0001 compared to the corresponding 5α-DHT values. ED_50_, dose causing 50% maximum effect; E_max_, maximum effect; DHT, dihydrotestosterone; Flut., flutamide; ns, non-significant. Data were analyzed using an unpaired *t*-test. Significant changes are highlighted in bold.

## Data Availability

The raw data supporting the conclusions of this article will be made available by the authors on request.

## Supplementary Materials

The following supporting information can be downloaded at: https://www.mdpi.com/article/10.3390/pharmaceutics18091063/s1.

## Abbreviations

The following abbreviations are used in this manuscript:

3α-HSD	3α-17β-hydroxysteroid dehydrogenase
3β-HSD	3β-17β-hydroxysteroid dehydrogenase
5α-DHT	5α-dihydrotestosterone
5β-DHT	5β-dihydrotestosterone
AR	Androgen Receptors
AUC	Area Under Curve
BK channels	Large-conductance calcium-activated potassium channel
cAMP	Cyclic adenosine monophosphate
CaSR	Calcium sensing receptor
cGMP	Cyclic guanosine monophosphate
Cmax	Maximum concentration
DMSO	Dimethyl sulfoxide
EC50	Concentration causing 50% maximum effect
ED50	Dose causing 50% maximum effect
ELISA	Enzyme-Linked Immunosorbent Assay
Emax	Maximum effect
ERK1/2	Extracellular Signal-Regulated Kinases 1 and 2
FSH	Follicle-Stimulating Hormone
GnRH	Gonadotropin-Releasing Hormone
GPCRs	G-protein-coupled receptors
GPER	G-protein estrogen receptor
GPRC6A	G-protein-coupled receptor class C, group 6, subtype A
IGFBP6	Insulin-Like Growth Factor-Binding Protein 6
i.p.	Intraperitoneal
LH	Luteinizing Hormone
L-NAME	N(ω)-nitro-L-arginine methyl ester
MAPK	Mitogen-Activated Protein Kinase
mR	Membrane Receptor
NOS	Nitric oxide synthase
PKC	Protein Kinase C
PLC/DAG/IP3	Phospholipase C/Triacylglycerol/Inositol triphosphate
SD	Standard deviation
SPRD	Sprague Dawley
T	Testosterone
t1/2	Half-life time
tmax	Time to maximum concentration
UGT	Uridine 5′-diphospho--glucuronyltransferase
ZIP9	Zinc Transporter 9
