# Supplementary material for: Non-Genomic Actions of Testosterone Metabolites on Uterine Contractility in Rats
Source: Pharmaceutics. 2026 Aug 26;18(9):1063. doi: 10.3390/pharmaceutics18091063 (PMC13609958; doi:10.3390/pharmaceutics18091063)
Supplement: Supplementary file 1 [file pharmaceutics-18-01063-s001.zip › pharmaceutics-4424591-supplementary.pdf]

	Non-pregnant			22-day pregnant	
**Solvent effect**	**First admin.**	**Second admin.**		**First admin.**	**Second admin.**
	13.8873	3.715507		18.16538	10.26791
	11.5953	11.76538		17.67942	9.343313
	-3.02439	4.743648		5.75839	7.925737
	7.8024	12.79067		-7.6526	0.21182
	3.7694	6.29019		2.50951	9.502858
**Time effect**	**Time-15**	**Time-30**		**Time-15**	**Time-30**
	9.26997	5.283351		0.711395	4.780842
	-13.2854	9.615931		0.777265	2.011873
	9.68306	7.84226		5.329305	4.815687
	2.24668	2.585631		18.52138	-2.82895
	1.30986	1.563958		-4.25304	-7.36765
	12.5202	-6.59572		-0.35938	14.59921

	Log 5α-DHT	-8	-7	-6	-5	-4	-3
Non-pregnant	5α-DHT	-6.6206	15.5108	25.7035	3.2159	66.9352	66.0751
		-2.7077	14.3892	26.3400	32.3505	67.5076	67.3714
		11.5685	20.1173	27.1262	33.9578	68.1906	67.3046
		13.5699	29.7746	32.6554	44.4779	56.6650	67.1561
		12.1676	28.2085	34.2169	29.5919	67.1709	66.1634
		3.0930	15.7806	24.6896	30.8158	67.4678	65.6116
		1.9524	17.2336	17.3520	22.4562	67.9258	65.0652
		4.2709	9.7430	17.5473	25.4118	60.2623	65.1927
		18.6316	21.9322	25.0813	34.3579	64.4009	65.7421
		13.3213	15.1114	11.8989	22.2282	60.9871	61.4694
		9.8500	20.3159	24.6502	31.9795	66.0235	66.8717
		8.5092	18.1281	35.3525	38.9366	66.0357	66.6589
		16.0012	21.2973	25.7080	15.1144	49.3541	63.0763
		6.2997	7.3804	9.0442	14.8075	59.0541	65.8416
		3.2092	12.9871	10.3715	24.3515	56.5718	67.2246
		1.0047	10.2956	7.2120	29.7864	59.4682	63.9917
		2.7438	11.8529	7.3284	32.7710	60.3804	66.2751
	5α-DHT + Flu -6	-4.0969	0.3855	-0.8106	0.0941	60.4903	64.8504
		10.3267	14.4057	13.8975	8.3036	66.5019	68.3450
		-3.3795	3.2744	2.7790	6.2709	54.2696	63.7273
		-1.1149	24.3292	27.7787	34.1627	61.7284	68.4375
		7.8691	8.1666	3.6444	-9.2019	61.9069	62.8620
		2.8727	10.7695	10.5144	7.4144	64.2017	65.8319
		5.5621	17.4898	11.5017	5.2703	67.5175	67.5969
		5.0601	21.1404	14.8540	11.1559	65.2560	66.8692
		20.6610	28.9567	29.4878	26.9796	63.1918	67.9679
		10.9074	17.0024	16.7503	13.8884	65.3327	67.4490
		9.6949	14.7118	8.0032	1.4799	23.5864	62.3401
		8.4188	13.2498	11.2620	21.8041	57.2237	65.9935
		8.6864	15.7366	14.5786	12.0133	63.4498	66.6979
		5.5435	16.2511	16.4698	16.6853	64.0080	68.0048
	5α-DHT + Biclutamide -6 M	6.0380	14.2736	3.7354	17.6544	65.3106	68.7234
		8.4015	8.3784	16.4370	-3.2739	66.2749	67.8356
		5.3114	10.7340	12.4100	11.9597	63.4803	66.1480
		7.4659	6.2841	7.9318	5.4945	61.8730	65.6043
		-15.6644	-27.8757	3.5707	8.6452	62.3542	66.7979
		2.4337	7.3360	8.0666	0.8241	67.8629	68.4631
		4.7214	12.2849	13.2756	7.7711	64.6154	68.8749
		23.5789	28.7715	29.2948	34.6188	62.5582	64.5842
		23.1907	28.2030	26.5148	30.6999	60.2735	62.9854
		33.3850	35.2732	28.7298	30.5864	58.5441	63.1481
		7.6368	14.1409	11.4939	14.3301	58.2090	63.0002
		17.9609	21.0986	16.7042	27.6771	63.6639	67.1459
		-2.2089	1.5858	0.9552	10.6610	59.8748	63.7719
		11.3829	16.1296	15.6933	19.6286	61.0242	64.9804
		30.2584	27.5208	22.1954	27.3766	64.6395	67.9110

	Log 5α-DHT	-8	-7	-6	-5	-4	-3
22-day pregnant	5α-DHT	3.3679	4.5061	13.6681	21.2190	57.4004	62.2384
		-0.6564	16.9769	8.7409	15.8042	63.8950	62.8309
		9.0924	23.4467	10.1016	13.3749	44.3340	61.8181
		-0.3550	18.1575	25.4684	36.7628	49.4117	63.6500
		-1.7493	4.3094	6.2660	4.2167	27.8715	52.6487
		-7.7573	5.3479	8.1811	16.9015	53.6286	66.9546
		-3.4334	3.2120	15.7279	27.2153	39.9044	61.2451
		5.9409	8.4429	17.3586	21.7708	36.0222	60.3977
		-5.0468	11.0128	13.7301	28.0683	40.9146	54.4204
		-6.1301	0.4847	6.6331	24.7079	26.8324	58.8881
		-7.2524	3.6509	17.5757	38.4298	51.8662	69.2481
		-3.0097	8.4597	18.7120	27.3137	47.5194	66.4901
		1.9548	15.3444	24.1428	29.3851	31.8943	47.8093
		11.6526	0.7184	17.2127	8.3446	40.9351	65.4938
		-0.8343	3.5878	8.4956	20.3172	56.0232	67.3884
		7.7024	16.5523	27.8887	25.8255	63.1174	61.6168
		9.7259	11.4589	21.8085	39.4306	47.0105	66.3894
		8.6365	21.1120	29.0273	26.6495	51.3766	63.9759
	5α-DHT + Flu -6	4.1158	12.6568	18.7507	22.2085	42.2632	55.2843
		-2.6771	3.0991	14.9360	23.0100	58.3598	65.3586
		-0.0516	10.0571	16.2543	20.2904	31.0925	54.1869
		9.2158	24.0298	30.8250	24.1834	33.8953	58.1268
		2.7562	17.2593	25.3121	41.7856	53.2611	66.2327
		4.1555	16.4993	26.1191	31.3700	40.8030	46.0068
		12.8016	23.8928	30.6153	35.9313	42.5028	56.7709
		9.7643	18.5702	20.2256	22.5928	40.3275	64.2084
		-0.0136	5.1254	17.7173	34.8802	47.5904	65.2210
		1.3088	7.2912	12.4918	19.8029	48.9417	53.9223
		-1.8224	11.7813	15.8324	12.4892	35.3429	49.3968
		12.3924	21.7123	26.6101	22.6571	41.0760	62.7807
	5α-DHT + Bicalutamide -6 M	6.4417	9.9977	18.2575	25.0092	32.6581	58.3175
		5.9802	13.3173	16.1786	25.1868	36.8116	58.2997
		1.1588	12.7446	15.9951	25.3720	36.6609	61.5399
		0.7456	7.2125	12.9670	15.7816	34.6517	52.8403
		1.4978	6.1040	19.4386	26.3590	33.8811	44.9637
		-0.9757	5.5850	15.6455	24.9690	40.7833	51.1091
		-7.7167	4.4060	23.7653	34.6149	44.6695	69.6780
		-2.3572	2.7522	5.4186	23.6987	41.1956	59.8002
		6.0889	8.8163	10.4353	23.6141	30.9871	47.0174
		3.0640	6.8677	15.3068	21.6074	32.3318	45.1535
		2.8606	7.0851	16.1030	29.1439	45.3630	62.4331
		3.8836	7.2934	17.9734	27.5943	42.1318	56.3118
		2.8090	9.7638	14.3507	28.0274	49.3130	69.4542
		6.3815	10.4669	23.8443	22.5352	37.3202	54.9245
		2.0288	4.7831	10.7388	27.1602	44.0576	60.0294

	Log 5β-DHT	-9	-8	-7	-6	-5	-4
Non-pregnant	5β-DHT	-8.1633	10.0607	22.9782	35.9634	50.5925	66.3568
		2.4366	15.4961	19.4325	16.8815	57.5557	68.9046
		-3.7002	7.5413	21.9388	34.4022	51.3322	65.4276
		-3.2572	11.8726	27.1308	37.2200	46.3168	64.4491
		-8.3292	14.6033	25.5312	33.0849	50.3906	66.5799
		-1.9372	15.9550	21.9990	33.3654	51.3757	65.6339
		-5.4201	12.5437	20.7681	35.0056	59.6735	69.4113
		-6.6583	16.8375	29.4305	37.1393	57.1775	63.6017
		0.3108	7.9093	18.9887	28.4213	52.1675	65.7792
		-1.0936	7.0120	18.5523	21.3778	53.6466	68.4535
		-7.4999	14.7725	24.6704	42.3867	54.3751	67.9483
		2.0133	11.6653	22.5983	36.4947	55.7760	62.7352
		3.3258	18.8578	27.0569	38.2558	54.3174	70.6381
		-7.3455	12.4583	22.8663	37.9120	54.3378	67.9162
		-2.2459	7.7940	12.1416	30.3377	48.5005	65.3389
		5.9092	12.0042	19.7959	26.1142	42.5294	66.4981
		-2.0252	18.2765	20.7637	32.4229	51.8988	66.2487
		-7.8701	20.4520	27.6863	34.0414	50.2346	61.4356
		-2.0875	8.8780	25.5750	33.9550	47.2436	66.7787
		-1.9531	12.5462	27.5948	35.7432	43.4403	64.4273
	5β-DHT + Flutamide -6 M	0.8311	3.2590	12.5579	21.2677	34.9631	66.2036
		-0.6894	9.8033	16.8574	22.1222	46.8400	68.3119
		3.9603	10.2641	15.6532	34.3442	54.9657	67.5539
		2.3070	12.0794	14.8855	26.8695	58.8531	61.4210
		8.6234	9.5834	17.4133	22.7426	47.6825	66.6408
		27.9317	14.5440	21.4233	31.6388	64.6741	67.1260
		10.4124	19.2417	23.1742	26.7707	58.1094	62.4344
		-2.3564	16.1851	24.9073	38.7363	57.3693	60.8645
		8.8414	21.5250	17.7512	18.0945	37.8793	56.0130
		-10.7373	10.2909	14.0805	24.4361	56.3746	64.6456
	5β-DHT + Bicalutamide -6 M	9.3660	15.2436	26.1142	41.5660	58.3090	67.3615
		8.5981	18.2463	23.9677	49.5447	56.3412	62.5649
		7.0734	17.5521	30.6112	35.5916	45.7953	63.2790
		3.6323	13.6535	23.0220	44.2178	57.7955	62.8222
		6.8671	17.8353	27.9768	49.5197	55.9044	62.5138
		13.9003	22.7024	25.5871	33.1334	45.7944	60.0259
		4.3220	17.3953	24.0130	46.2865	50.6206	60.6826
		7.8918	19.0832	23.1674	46.1512	56.9054	68.4807
		5.2635	15.0332	21.7147	45.2814	56.0651	65.7014
		4.0018	19.8578	28.1884	40.7046	51.1092	63.1237
		13.8369	23.1530	33.9391	42.0000	51.2026	63.7119
		14.7741	17.4840	24.6243	34.3636	50.3905	66.6920
		-0.5152	17.4618	24.9692	37.0088	49.6872	64.9365
		-6.4122	12.5554	24.7414	43.4982	54.2851	65.3817

	Log 5β-DHT	-9	-8	-7	-6	-5	-4
22-day pregnant	5β-DHT	7.0639	18.2448	28.9618	31.7708	52.1884	55.2065
		5.2938	22.6135	28.1828	21.7643	59.8755	60.9460
		3.5695	5.1445	24.9582	33.8380	49.3795	51.6203
		3.5427	8.4930	28.1391	34.3702	59.0562	58.7945
		2.5616	15.5771	26.6427	36.0038	60.6980	62.4707
		1.8272	7.6778	28.1762	37.9670	56.2701	58.1000
		8.6391	18.2627	22.8685	38.3102	50.4705	52.2585
		2.8124	10.4433	28.8462	38.4410	51.3107	55.4068
		2.1660	16.1332	24.9106	32.6364	41.9808	50.2910
		6.3804	14.6519	25.4444	36.1122	52.7803	54.7167
		1.9275	15.3873	28.3821	36.6721	45.5175	53.4643
		3.5427	8.4930	28.1391	36.3063	42.0562	50.7945
		-0.9304	15.1524	17.1953	42.1342	59.5817	53.2921
		13.3534	28.4221	29.2153	47.8987	54.2068	55.5180
		25.2623	37.4950	36.4369	47.2734	59.0393	68.6795
		12.0628	9.6309	12.0532	22.4331	55.5976	59.4416
		10.7534	21.3467	29.0085	35.1269	42.4644	50.3217
		17.8220	25.1583	27.5993	34.7910	47.2678	62.3717
		12.5181	25.2459	33.5786	32.6464	48.2860	67.8727
		19.7409	33.0255	33.8028	38.7723	49.9581	64.0113
	5β-DHT + Flutamide -6 M	8.9620	20.5690	34.5211	45.6743	65.3522	66.9461
		13.1498	26.8574	35.0159	38.9481	60.4962	64.7822
		17.7378	29.9033	34.5117	49.8258	58.5825	61.7402
		11.1063	24.5396	35.9767	45.1148	69.8585	70.1504
		13.0630	24.7831	33.8823	45.9598	51.5801	63.7976
		10.9124	21.8904	30.6713	48.7233	63.3439	65.1416
		18.5243	29.8742	41.6189	48.8628	64.0581	67.6705
		14.5264	19.1750	38.1061	46.5498	58.9607	60.4699
		16.4145	21.2224	37.6982	51.9757	55.9981	60.4349
		11.7264	20.0163	38.2040	50.1051	65.7714	66.5716
		19.6221	25.1056	41.4622	46.5297	56.4554	62.9721
		13.4363	24.4125	32.0079	50.5185	59.3654	63.8185
		13.7673	22.6592	36.1024	30.6838	43.0364	55.3170
		19.5697	23.2987	39.4576	45.1022	64.3582	66.9669
		19.1353	27.6102	36.7231	48.8224	60.5306	64.9474
		20.0935	25.7564	32.4243	37.0413	43.7418	57.5999
	5β-DHT + Bicalutamide -6 M	3.1134	5.8113	23.8452	46.1035	53.5509	67.7833
		-1.3188	6.6805	22.9977	48.2644	48.2743	63.1296
		12.1185	31.2450	37.7450	40.4329	52.1804	60.1466
		13.0006	22.1007	32.1721	48.3204	55.5565	61.6558
		10.9061	23.9237	31.0188	36.2306	42.6742	64.5767
		14.4386	24.0945	30.0713	33.3323	45.5017	59.2112
		14.1441	26.9447	31.8604	40.7861	51.7284	55.4943
		2.7036	14.7550	30.1165	42.0199	63.0215	67.9244
		9.3070	23.6211	32.1406	36.2468	58.7483	63.2240
		16.3149	28.6909	33.3790	48.9580	57.8662	61.0784
		5.5142	19.7925	31.9491	41.6620	67.9623	68.2726
		8.6656	20.9786	30.5380	43.2264	49.1309	61.9664
		7.7487	19.1167	28.2094	46.9024	62.0422	68.0458
		1.8133	15.4912	29.6448	38.3743	56.6862	65.5008
		2.5957	7.8932	29.4667	39.0891	53.2324	66.3480

	Log 5α-DHT	-8	-7	-6	-5	-4	-3
Non-pregnant rats	5α-DHT	-6.6206	15.5108	25.7035	3.2159	66.9352	66.0751
		-2.7077	14.3892	26.3400	32.3505	67.5076	67.3714
		11.5685	20.1173	27.1262	33.9578	68.1906	67.3046
		13.5699	29.7746	32.6554	44.4779	56.6650	67.1561
		12.1676	28.2085	34.2169	29.5919	67.1709	66.1634
		3.0930	15.7806	24.6896	30.8158	67.4678	65.6116
		1.9524	17.2336	17.3520	22.4562	67.9258	65.0652
		4.2709	9.7430	17.5473	25.4118	60.2623	65.1927
		18.6316	21.9322	25.0813	34.3579	64.4009	65.7421
		13.3213	15.1114	11.8989	22.2282	60.9871	61.4694
		9.8500	20.3159	24.6502	31.9795	66.0235	66.8717
		8.5092	18.1281	35.3525	38.9366	66.0357	66.6589
		16.0012	21.2973	25.7080	15.1144	49.3541	63.0763
		6.2997	7.3804	9.0442	14.8075	59.0541	65.8416
		3.2092	12.9871	10.3715	24.3515	56.5718	67.2246
		1.0047	10.2956	7.2120	29.7864	59.4682	63.9917
		2.7438	11.8529	7.3284	32.7710	60.3804	66.2751
	5α-DHT + Mife -6	13.4865	22.3270	28.3051	32.7942	62.6100	68.5050
		21.2520	25.0952	16.7857	28.2370	64.6270	68.0378
		7.6956	7.5474	12.4470	14.7792	67.0092	67.2899
		-0.9828	4.1647	11.3882	23.1834	60.8529	67.8545
		20.3295	31.0652	29.9453	20.5869	64.5707	69.3107
		14.9020	19.5623	15.9402	14.7904	68.0800	68.4770
		2.8037	9.1065	8.6955	12.5717	59.2520	67.9125
		3.9720	5.5047	10.6881	33.5218	53.4614	68.0958
		26.0910	39.0442	33.3365	38.7568	57.7973	63.1774
		8.8380	20.0981	14.6318	15.0746	66.1208	66.2588
		2.7922	30.5202	18.6524	14.5237	66.4917	67.0027
		-0.5090	14.9740	23.9537	34.3412	65.9072	65.9598
		13.0513	22.7673	17.5873	22.0172	59.7686	65.9990
		-9.6321	8.0628	2.0552	4.9126	51.8298	65.8327
		10.7937	22.4811	24.1562	7.1812	53.3225	62.4785
	5α-DHT + G15 -6 M	11.7932	13.3286	15.3417	23.1573	61.4191	57.7463
		13.2879	26.1932	31.7187	34.0140	57.4169	56.0363
		16.9612	23.9832	24.3312	20.8275	55.0945	58.8250
		6.4358	16.8956	26.5745	35.0220	45.6452	64.6169
		14.9649	20.8300	14.5960	34.7218	66.5303	67.1630
		10.1518	12.9782	12.9614	23.2943	48.6558	54.7711
		6.4997	14.2050	11.9914	23.0210	47.0685	62.8677
		26.8983	39.1844	33.1471	40.6730	59.9576	60.5471
		18.6650	32.2100	35.6886	23.2080	53.8523	60.9034
		32.8751	36.4648	32.2848	39.3406	40.6260	58.4453
		21.3895	33.5144	18.0591	27.2079	44.7793	65.4237
		14.0379	18.6254	18.5452	21.2953	45.1199	67.1971
		7.9821	18.9758	25.9393	30.4468	67.0719	67.9225
		29.6935	22.5448	17.2459	23.1078	61.8580	64.0571

	Log 5α-DHT	-8	-7	-6	-5	-4	-3
**22-day pregnant rats**	**5α-DHT**	3.3679	4.50612	13.66806	21.21897	57.4004	62.23839
		-0.65642	16.97687	8.74088	15.80418	63.89503	62.83093
		9.09236	23.44673	10.1016	13.37486	44.33402	61.81806
		-0.35497	18.15748	25.46835	36.76276	49.41174	63.64997
		-1.74926	4.30936	6.26599	4.21672	27.87147	52.64874
		-7.7573	5.34792	8.18114	16.90145	53.62864	66.95464
		-3.4334	3.21204	15.72791	27.21528	39.90439	61.24506
		5.94087	8.44288	17.35859	21.7708	36.02216	60.39771
		-5.04681	11.01277	13.73011	28.0683	40.91461	54.42041
		-6.1301	0.48469	6.63307	24.70792	26.83244	58.88805
		-7.25242	3.65091	17.57565	38.42983	51.86624	69.24811
		-3.00972	8.45968	18.71203	27.31366	47.51938	66.49011
		1.95481	15.34435	24.14284	29.3851	31.89428	47.80929
		11.65257	0.71838	17.21265	8.34463	40.93513	65.49382
		-0.83427	3.58782	8.49563	20.31718	56.02323	67.38843
		7.702403	16.55234	27.88867	25.82554	63.11739	61.61675
		9.72585	11.45891	21.80851	39.43064	47.01052	66.38935
		8.63653	21.11201	29.02733	26.64946	51.37659	63.97592
	**5α-DHT + Mife -6**	5.941668	21.0875	27.34375	32.2223	50.159	49.28131
		3.97856	13.07151	35.35515	37.02707	46.57258	55.8439
		-2.75246	7.19523	22.0618	28.73143	44.46025	65.06857
		-1.67156	-1.77108	23.65912	30.48136	34.43907	51.50649
		-5.10802	9.2074	19.91918	28.95605	32.74481	46.69176
		12.82813	20.58868	27.88415	27.72933	46.97727	54.23482
		-1.5747	6.85059	14.90808	23.2481	38.17907	56.46038
		-6.72084	6.848726	9.63783	22.32521	42.37865	68.57535
		-1.09679	6.897456	15.90262	29.11153	50.52823	69.72555
		-3.8779	15.7163	20.2906	24.7068	45.41265	60.98515
		-8.29329	6.9124	13.87038	17.923	49.51549	64.11517
	**5α-DHT + G15 -6 M**	-1.70939	8.55955	22.75575	20.68357	29.24163	51.29595
		-13.2724	-3.61432	14.16237	9.00472	28.01995	50.89359
		-6.91585	1.906158	15.92864	8.23286	22.20364	44.41926
		0.157483	11.35635	30.25291	30.39625	48.24892	64.59239
		19.06703	34.02015	47.81377	44.23294	51.07767	59.93922
		6.112672	14.19606	13.92281	23.06987	30.07811	50.86015
		6.19736	13.8695	25.51053	36.39	47.23942	63.67486
		0.731672	6.482262	13.56953	22.40509	45.44296	56.59329
		14.022	24.01099	25.78738	27.51684	35.6516	51.33622
		7.414546	15.05523	19.43277	11.85284	26.42532	45.78679
		4.588949	17.96891	20.5258	15.91874	23.82339	49.41169
		13.08619	33.50181	40.36939	39.43316	50.82098	67.32545
		7.642495	16.86454	23.7376	22.22292	31.12121	54.77368
		5.733559	19.75046	26.50359	24.68528	38.833	59.68315

	Log 5β-DHT	-9	-8	-7	-6	-5	-4
Non-pregnant rats	5β-DHT	-8.1633	10.06067	22.97819	35.96335	50.59245	66.35678
		2.436611	15.49607	19.43247	16.88152	57.55571	68.9046
		-3.70023	7.5413	21.93878	34.40224	51.3322	65.42762
		-3.25715	11.87262	27.1308	37.21998	46.31684	64.44911
		-8.3292	14.60328	25.53124	33.08488	50.39057	66.57987
		-1.93724	15.95504	21.99898	33.3654	51.3757	65.63386
		-5.42013	12.5437	20.7681	35.00558	59.67354	69.41128
		-6.65827	16.83748	29.43047	37.13926	57.1775	63.60165
		0.310816	7.90927	18.98865	28.42126	52.16752	65.77921
		-1.0936	7.01199	18.55233	21.37778	53.64659	68.45351
		-7.4999	14.77249	24.6704	42.3867	54.37507	67.94833
		2.013316	11.66532	22.59825	36.4947	55.77597	62.73521
		3.325814	18.85778	27.05691	38.25581	54.31738	70.63808
		-7.3455	12.4583	22.8663	37.912	54.33778	67.91615
		-2.24591	7.79403	12.14156	30.33774	48.50053	65.33888
		5.909246	12.00419	19.7959	26.11417	42.52935	66.49807
		-2.02524	18.27645	20.76372	32.42288	51.89879	66.2487
		-7.87014	20.452	27.6863	34.0414	50.2346	61.43555
		-2.0875	8.87796	25.575	33.95501	47.24357	66.77871
		-1.9531	12.54616	27.59477	35.74315	43.44033	64.42729
	5β-DHT+ Mifepristone -6 M	7.26587	10.48	16.2719	25.8371	44.62852	68.46974
		0.675716	1.354017	11.52814	11.86732	44.44716	67.74053
		-6.36693	12.8617	17.90482	20.93229	41.84462	67.43933
		-8.6396	11.0288	26.3745	30.0614	44.403	58.25619
		-4.3695	10.834	19.35253	22.80542	42.20563	51.34969
		-5.3797	14.16825	27.03791	36.80633	45.50328	65.36247
		3.982543	13.48626	29.17183	34.25048	52.88871	67.90417
		-4.09453	3.343033	19.98866	42.60286	51.3591	65.05074
		-10.7599	6.1487	20.42472	38.1565	43.9896	68.50703
		2.74726	14.73282	16.87316	38.79508	55.56373	66.04591
		7.25806	10.73051	34.04369	43.97459	58.68449	67.30062
		4.51082	13.49025	28.08497	31.90624	50.85613	66.8449
		7.486418	9.61534	23.56297	21.74932	43.81404	66.69758
		-8.50967	3.13598	15.13964	29.50774	45.08726	65.80479
		0.023101	7.75968	15.78227	31.90854	50.43577	68.11282
		-1.59892	11.53725	20.50238	16.30044	50.638	67.55181
		-4.55343	12.38628	15.51574	29.07335	39.54017	58.91828
		-1.3367	3.61341	15.83252	22.82753	41.68553	50.59457
		-15.2494	7.27539	13.71649	28.71307	36.57374	53.95757
	5β-DHT + G15 -6M	7.36224	19.96441	23.62424	32.70578	51.23506	62.74343
		3.74261	19.98983	25.98676	35.04517	49.91686	67.40635
		7.095418	13.20181	16.0208	23.84807	43.75819	65.9907
		4.11242	14.41614	21.69325	39.75465	46.17074	67.4378
		3.323791	19.95163	14.71611	34.55606	40.7154	68.88445
		-3.44468	17.73846	23.6945	42.73932	57.09869	66.79111
		7.62753	15.39072	20.08534	36.24772	41.86129	68.09597
		7.33215	20.30246	26.06871	10.08775	54.97691	68.09321
		5.716128	7.34125	10.64646	21.34022	35.836	65.12873
		17.33691	13.50003	20.06788	7.05148	30.52402	65.65042
		13.91494	20.33301	22.83443	9.4202	35.74084	68.599
		5.805112	9.01769	3.22346	-1.14742	36.16878	68.45536

	Log 5β-DHT	-9	-8	-7	-6	-5	-4
22-day pregnant	5β-DHT	7.06385	18.24483	28.96179	31.77075	52.18843	55.20654
		5.293839	22.61353	28.18282	21.76425	59.87547	60.94604
		3.569544	5.144506	24.95818	33.83798	49.37951	51.62027
		3.542728	8.493027	28.1391	34.37024	59.05624	58.79446
		2.561642	15.57709	26.64274	36.00384	60.698	62.47071
		1.82721	7.677819	28.17618	37.96701	56.27013	58.10002
		8.639111	18.2627	22.86845	38.3102	50.47051	52.25847
		2.812435	10.44325	28.84619	38.44099	51.31072	55.40682
		2.165981	16.13321	24.91061	32.63641	41.9808	50.29098
		6.380361	14.6519	25.44435	36.11222	52.78031	54.71674
		1.927498	15.38731	28.38206	36.67207	45.51752	53.46431
		3.542728	8.493027	28.1391	36.30629	42.05624	50.79446
		-0.93035	15.15241	17.19528	42.13419	59.58169	53.29205
		13.35338	28.42212	29.21531	47.89871	54.20676	55.51801
		25.2623	37.49501	36.43685	47.27343	59.0393	68.67946
		12.06275	9.630905	12.05315	22.43308	55.59756	59.44163
		10.75339	21.34665	29.00849	35.12692	42.46441	50.32174
		17.82197	25.15829	27.59928	34.79097	47.2678	62.37174
		12.51813	25.24585	33.57863	32.64639	48.28602	67.87272
		19.74091	33.02549	33.8028	38.7723	49.95814	64.01126
	5β-DHT+ Mifepristone -6 M	-7.65896	12.61758	6.507175	18.0325	44.59805	62.91424
		-17.1761	-4.73302	6.072554	24.86279	35.34385	68.34897
		-9.82272	-3.91173	-3.23544	4.217712	27.26139	51.09327
		-13.5416	-0.34793	7.658591	22.07123	41.26913	67.67069
		7.663109	10.56436	20.16175	38.8416	61.64491	69.60428
		6.889624	16.69952	27.42022	39.39035	52.84036	64.61326
		4.986939	16.85115	32.28099	37.06334	48.68546	68.13735
		4.519627	7.069966	19.47479	35.15905	53.80612	67.82742
		2.698954	14.48297	23.39278	38.744	58.59698	67.23146
		7.023665	16.89694	25.4709	30.05424	52.82745	61.46158
		7.604782	12.74606	23.08378	53.09098	63.90803	65.76211
		5.434077	16.42274	23.09117	35.09509	51.08454	68.05998
	5β-DHT + G15 -6 M	-0.61111	7.232189	10.07671	16.88751	34.06952	50.1765
		6.341189	14.28724	18.06355	23.89175	28.72586	54.41643
		-5.46764	1.355913	17.06117	20.51048	41.37409	56.39177
		13.77521	22.83116	26.51685	30.47747	45.06927	53.32772
		-1.01493	2.792203	14.50632	16.75886	33.22731	53.98522
		4.657563	10.00081	23.36844	29.56741	38.56072	60.03149
		-7.14163	8.89713	17.55193	23.44965	42.29172	58.53095
		9.423473	13.79433	15.75596	24.1524	39.07198	57.14548
		5.032186	12.43555	15.12053	21.5493	30.10741	52.97111
		0.863498	7.277441	22.64021	38.70238	44.09784	64.40925
		4.232703	7.842057	19.46713	28.09628	54.61851	56.37564
		8.729514	14.049	16.45924	34.6813	40.09739	53.19253

	Log 5α–DHT	-8	-7	-6	-5	-4	-3
Non-prenant rats	5α-DHT	-6.62059	15.5108	25.7035	3.21587	66.9352	66.0751
		-2.70766	14.3892	26.34	32.3505	67.5076	67.3714
		11.5685	20.1173	27.1262	33.9578	68.1906	67.3046
		13.5699	29.7746	32.6554	44.4779	56.665	67.1561
		12.1676	28.2085	34.2169	29.5919	67.1709	66.1634
		3.09302	15.7806	24.6896	30.8158	67.4678	65.6116
		1.95236	17.2336	17.352	22.4562	67.9258	65.0652
		4.27085	9.743	17.5473	25.4118	60.2623	65.1927
		18.6316	21.9322	25.0813	34.3579	64.4009	65.7421
		13.3213	15.1114	11.8989	22.2282	60.9871	61.4694
		9.84998	20.3159	24.6502	31.9795	66.0235	66.8717
		8.50924	18.1281	35.3525	38.9366	66.0357	66.6589
		2.74378	11.8529	7.32836	32.771	60.3804	66.2751
		16.0012	21.2973	25.708	15.1144	49.3541	63.0763
		6.29965	7.38042	9.04423	14.8075	59.0541	65.8416
		3.20916	12.9871	10.3715	24.3515	56.5718	67.2246
		1.00467	10.2956	7.21198	29.7864	59.4682	63.9917
	5α-DHT+ endometrial remova	8.02653	21.7186	36.9147	40.3185	54.4109	56.4781
		8.28355	19.1576	20.0998	34.6288	63.676	64.3666
		11.614	20.2113	30.0424	9.55474	44.3943	61.2801
		3.03698	10.0762	27.3434	40.0368	64.8402	64.7345
		34.3803	40.7913	24.8438	23.1488	56.1101	64.4231
		5.18869	7.51695	10.1351	19.6657	65.8965	62.6034
		12.9054	22.8679	19.0439	18.8125	55.1774	62.7894
		12.5663	15.5754	14.9628	8.2432	65.5583	65.3821
		15.2135	22.1352	18.1919	13.0611	52.4432	61.4087
		9.01906	12.357	19.1331	25.9754	66.6795	65.8831
		16.0153	21.6192	26.9114	24.6494	44.4286	48.509
		13.7378	21.4948	17.1939	27.5571	58.4449	58.0082
		-1.23808	18.7419	17.4861	12.0037	63.0783	60.0363
	5α-DHT + L-NAME -5M	1.00734	16.2555	16.4796	17.3263	60.2046	65.8152
		-4.69244	4.91045	18.5539	23.285	40.3062	68.9112
		16.8613	59.1631	44.1184	36.6358	51.3484	67.1932
		9.0446	21.281	24.9281	24.8925	55.7768	66.7733
		3.06376	12.2047	11.6931	5.70033	44.1452	63.7987
		-0.17016	24.4682	33.3226	43.2543	57.8287	63.1455
		11.4711	18.3625	13.4422	18.5692	52.3904	65.7786
		9.80105	11.019	14.9668	22.2963	37.3937	62.4291
		-2.84847	7.45331	4.71775	33.7966	60.483	67.1407
		0.10803	6.75953	7.4493	0.62618	50.6206	66.217
		3.06439	8.23154	19.4632	22.3283	45.1523	67.379
		16.5282	25.2297	27.6932	19.4215	50.7935	64.4563

	Log 5α–DHT	-8	-7	-6	-5	-4	-3
22-day pregnant rats	5α-DHT	3.3679	4.50612	13.6681	21.219	57.4004	62.2384
		-0.65642	16.9769	8.74088	15.8042	63.895	62.8309
		9.09236	23.4467	10.1016	13.3749	44.334	61.8181
		-0.35497	18.1575	25.4684	36.7628	49.4117	63.65
		-1.74926	4.30936	6.26599	4.21672	27.8715	52.6487
		-7.7573	5.34792	8.18114	16.9015	53.6286	66.9546
		-3.4334	3.21204	15.7279	27.2153	39.9044	61.2451
		5.94087	8.44288	17.3586	21.7708	36.0222	60.3977
		-5.04681	11.0128	13.7301	28.0683	40.9146	54.4204
		-6.1301	0.48469	6.63307	24.7079	26.8324	58.8881
		-7.25242	3.65091	17.5757	38.4298	51.8662	69.2481
		-3.00972	8.45968	18.712	27.3137	47.5194	66.4901
		1.95481	15.3444	24.1428	29.3851	31.8943	47.8093
		11.6526	0.71838	17.2127	8.34463	40.9351	65.4938
		-0.83427	3.58782	8.49563	20.3172	56.0232	67.3884
		7.7024	16.5523	27.8887	25.8255	63.1174	61.6168
		9.72585	11.4589	21.8085	39.4306	47.0105	66.3894
		8.63653	21.112	29.0273	26.6495	51.3766	63.9759
	α-DHT + Endometrial Remova	-3.96475	9.02166	18.6224	11.4114	42.4032	56.9696
		1.49699	11.2162	17.6201	12.159	43.3258	64.5468
		4.12378	10.3103	17.0271	28.608	45.6614	66.6803
		4.83113	15.8233	20.9991	37.7536	45.3143	53.5133
		5.87535	18.3457	19.6743	26.5055	41.4895	67.3634
		-4.04336	2.46736	15.1573	23.6256	44.4901	68.7144
		-7.40777	5.17706	9.71586	11.2322	37.1822	56.5165
		2.16047	11.7174	19.3203	19.939	32.7301	53.4822
		1.23064	13.8808	16.927	28.7637	40.2448	62.6618
		-1.88551	11.481	18.8291	23.0459	48.545	60.9357
		-4.3891	8.28234	17.4233	18.5543	34.9742	52.3963
	5α-DHT + L-NAME -5M	2.41419	8.2485	15.974	31.3578	50.8947	69.7997
		7.22124	13.6348	15.1785	31.7862	54.4376	63.512
		5.79804	17.5865	27.5264	34.6362	54.07	63.8102
		7.31329	19.9134	33.1357	46.7144	52.6199	67.7162
		6.54846	8.73936	16.6593	22.7098	39.6236	50.133
		0.78591	5.36173	6.11126	-1.35486	31.2763	59.8837
		15.9162	24.645	23.1071	15.2915	27.5938	48.3694
		10.6234	19.2091	19.0164	25.6001	42.2044	59.0259
		20.9413	8.93791	9.22003	20.3954	43.7642	62.8937
		6.48149	9.29659	18.5534	28.4601	36.1761	57.1879
		-13.4346	-4.73167	0.58316	-2.40644	32.3092	51.5361
		-6.64483	1.06142	11.1088	6.72848	29.975	50.284
		9.50445	21.611	16.7343	9.04002	27.9293	47.0585
		-0.2208	20.1004	18.8629	5.62829	32.415	52.5172
		7.93518	12.1333	24.0412	17.0143	25.7601	51.89

	Log 5β-DHT	-9	-8	-7	-6	-5	-4
		-3.70023	7.5413	21.9388	34.4022	51.3322	65.4276
		-8.1633	10.0607	22.9782	35.9634	50.5925	66.3568
		2.43661	15.4961	19.4325	16.8815	57.5557	68.9046
		-3.25715	11.8726	27.1308	37.22	46.3168	64.4491
		-8.3292	14.6033	25.5312	33.0849	50.3906	66.5799
		-1.93724	15.955	21.999	33.3654	51.3757	65.6339
		-5.42013	12.5437	20.7681	35.0056	59.6735	69.4113
		-6.65827	16.8375	29.4305	37.1393	57.1775	63.6017
		0.31082	7.90927	18.9887	28.4213	52.1675	65.7792
	5β-DHT	-1.0936	7.01199	18.5523	21.3778	53.6466	68.4535
		-7.4999	14.7725	24.6704	42.3867	54.3751	67.9483
		2.01332	11.6653	22.5983	36.4947	55.776	62.7352
		3.32581	18.8578	27.0569	38.2558	54.3174	70.6381
		-7.3455	12.4583	22.8663	37.912	54.3378	67.9162
		-2.24591	7.79403	12.1416	30.3377	48.5005	65.3389
		5.90925	12.0042	19.7959	26.1142	42.5294	66.4981
		-2.02524	18.2765	20.7637	32.4229	51.8988	66.2487
		-7.87014	20.452	27.6863	34.0414	50.2346	61.4356
		-2.0875	8.87796	25.575	33.955	47.2436	66.7787
		-1.9531	12.5462	27.5948	35.7432	43.4403	64.4273
Non-prenant rats		-1.02479	5.78143	39.5715	44.3424	54.1827	68.5736
		1.16443	10.9643	26.8952	31.3009	48.3986	66.5306
		-6.0322	14.1367	28.5692	30.9289	43.1842	63.098
		-4.69939	3.96544	12.6219	28.5605	34.0251	59.865
		0.29359	13.9324	24.2004	30.6661	42.4629	70.104
	5β-DHT+ Endometrial Remova	0.9093	7.80185	17.6847	27.509	41.5442	66.8296
		-5.23019	0.35772	5.46697	11.3527	38.2083	67.0052
		-4.49733	14.5548	14.6707	16.9938	42.534	61.4192
		3.153	11.9275	8.71139	17.3618	55.822	68.2738
		-7.89784	0.67944	12.5346	31.4978	45.0373	68.1893
		-11.417	15.8191	33.3257	42.7379	52.8091	52.0426
		11.4505	5.20485	7.4366	24.7165	34.3584	58.5102
		12.3163	6.57684	12.546	26.3722	45.1007	66.8039
		3.61759	13.4898	9.64332	23.4244	47.1393	68.0605
		1.96368	15.4802	7.4509	22.5143	48.3027	66.3637
	5β-DHT+ L-NAME -5M	9.87563	8.2252	12.6884	29.2276	39.9282	64.6911
		2.61912	15.1308	18.7956	25.2354	45.0087	67.4059
		3.48038	13.1285	10.6409	33.1058	46.4734	65.2289
		9.72912	12.8168	15.8133	30.8013	41.4835	65.2441
		1.91014	5.39145	14.3218	21.236	44.8891	66.7139
		-0.16975	6.66285	8.97587	25.5825	49.5461	63.8675

	Log 5β-DHT	-9	-8	-7	-6	-5	-4
22-day pregnant rats	5β-DHT	7.06385	18.2448	28.9618	31.7708	52.1884	55.2065
		5.29384	22.6135	28.1828	21.7643	59.8755	60.946
		3.56954	5.14451	24.9582	33.838	49.3795	51.6203
		3.54273	8.49303	28.1391	34.3702	59.0562	58.7945
		2.56164	15.5771	26.6427	36.0038	60.698	62.4707
		1.82721	7.67782	28.1762	37.967	56.2701	58.1
		8.63911	18.2627	22.8685	38.3102	50.4705	52.2585
		2.81244	10.4433	28.8462	38.441	51.3107	55.4068
		2.16598	16.1332	24.9106	32.6364	41.9808	50.291
		6.38036	14.6519	25.4444	36.1122	52.7803	54.7167
		1.9275	15.3873	28.3821	36.6721	45.5175	53.4643
		3.54273	8.49303	28.1391	36.3063	42.0562	50.7945
		-0.93035	15.1524	17.1953	42.1342	59.5817	53.2921
		13.3534	28.4221	29.2153	47.8987	54.2068	55.518
		25.2623	37.495	36.4369	47.2734	59.0393	68.6795
		12.0628	9.63091	12.0532	22.4331	55.5976	59.4416
		10.7534	21.3467	29.0085	35.1269	42.4644	50.3217
		17.822	25.1583	27.5993	34.791	47.2678	62.3717
		12.5181	25.2459	33.5786	32.6464	48.286	67.8727
		19.7409	33.0255	33.8028	38.7723	49.9581	64.0113
	5β-DHT+Endometrial Remova	6.43208	9.55509	26.2156	28.5583	42.1705	63.0095
		4.5334	5.32452	29.235	30.9598	59.1035	60.4209
		0.4561	2.3569	23.5327	27.8776	49.6837	71.6588
		-4.08565	1.80504	19.1005	32.3826	44.7622	59.0876
		-4.38399	2.88333	16.4204	29.3549	38.9901	50.1395
		3.67353	5.14602	23.001	37.6644	59.6026	63.1021
		5.51482	6.04098	20.3678	36.8428	47.0444	48.8094
		1.82572	14.4874	22.9572	33.8103	46.1257	66.581
		2.28577	5.52661	21.4985	36.3952	39.7117	59.1647
		8.00409	13.93003	25.5563	30.0889	44.7326	54.3831
		1.51708	7.99446	12.5194	29.8952	39.3206	54.1118
		3.84446	10.715	14.4514	21.3806	42.4519	61.5538
		-3.4427	15.4869	18.8974	28.6679	34.4033	62.2296
		-3.40662	4.41638	12.4739	13.9924	33.2907	59.581
	5β-DHT+ L-NAME -5M	2.21494	6.73938	8.3943	13.8926	25.8051	64.3911
		5.43281	10.6012	15.6491	20.6584	33.192	55.7563
		3.91133	8.87676	11.4557	25.1994	34.5088	51.3136
		2.79158	10.1153	12.023	24.6932	41.1789	56.1263
		-3.80835	1.94673	9.9094	13.7941	28.1563	63.8691
		0.079	10.9582	15.9858	16.3851	27.0058	51.1393
		7.86856	16.217	22.3929	35.4282	48.8426	67.5766
		4.99114	13.1445	20.3738	31.3091	40.2924	58.691
		3.49502	17.6075	24.6548	36.4526	45.2386	56.4359
		16.7865	21.5018	35.2396	41.0459	50.1797	62.0556
		8.03496	11.7708	22.0037	32.4448	47.3167	52.4723
		3.25466	15.6362	33.4028	47.4463	54.9279	67.6404
		4.50511	10.9614	25.8914	34.9795	47.5243	58.4347
		15.4268	26.7172	35.0163	35.8963	63.6076	64.0161

	Log 5α-DHT	-8	-7	-6	-5	-4	-3
Non-pregnant rats	5α-DHT	-6.62059	15.51079	25.70345	3.21587	66.93515	66.07506
		-2.70766	14.38916	26.34004	32.35053	67.50756	67.37138
		11.5685	20.11731	27.12616	33.95784	68.19055	67.30455
		13.56994	29.77457	32.65538	44.47792	56.66504	67.15605
		12.16756	28.20854	34.21692	29.59189	67.17094	66.16336
		3.093024	15.78059	24.68963	30.8158	67.46784	65.61157
		1.952364	17.23363	17.35203	22.45619	67.92584	65.06518
		4.27085	9.743	17.54734	25.41181	60.2623	65.19265
		18.63157	21.93222	25.08125	34.35793	64.40086	65.7421
		13.32134	15.11142	11.89894	22.22817	60.98712	61.46935
		9.84998	20.31594	24.65023	31.97946	66.02354	66.87168
		8.50924	18.12809	35.3525	38.93656	66.03571	66.65893
		2.743779	11.85292	7.32836	32.771	60.38037	66.27508
		16.00121	21.29725	25.70803	15.1144	49.3541	63.07632
		6.29965	7.38042	9.04423	14.80745	59.05413	65.84158
		3.209161	12.9871	10.37152	24.35148	56.57179	67.22462
		1.00467	10.29557	7.21198	29.78636	59.46822	63.99171
	5α-DHT + Paxilline -5 M	2.094804	18.00772	23.6301	27.18401	66.22983	64.62473
		-1.30777	3.245044	22.52584	27.71934	56.18138	67.89472
		-7.00909	7.54089	13.50833	32.82633	66.49752	66.74254
		-7.16121	2.27925	20.44926	30.47688	63.92485	63.03213
		11.93706	24.00447	21.81596	36.9628	65.93	62.81233
		-10.1319	4.84566	15.20783	36.51571	64.01574	62.93085
		5.011169	14.54266	17.64461	23.88519	55.09248	58.73352
		0.862169	9.95973	3.65464	11.95517	67.46959	66.14503
		8.30594	21.80464	17.49757	17.24378	56.42784	57.93882
		13.91381	6.27488	12.88211	27.00273	59.18779	53.12308
		10.54338	21.45238	22.20051	19.38514	44.27099	57.40758
		14.03501	20.90791	37.51682	52.03676	65.6992	65.70575
		-0.1178	9.54334	9.26172	12.05981	57.59603	55.19177
		12.58381	15.3986	17.87387	41.99182	64.89513	64.2108
	5α-DHT + NPS -5M	-4.81315	15.6283	20.93824	38.66758	65.76633	67.03752
		10.95239	15.5435	28.78932	56.18302	65.5379	65.99396
		8.53482	13.02027	24.86594	63.50685	62.67741	62.47527
		-0.86139	18.17093	18.90522	31.89836	64.98914	63.7676
		13.57577	15.07197	30.10407	48.34079	62.12595	65.05516
		9.99154	13.56062	26.98403	59.84629	61.20739	63.11325
		5.29526	12.23589	24.02936	31.30472	63.24773	64.36472
		19.04472	29.62941	34.29499	37.71592	58.08403	60.68818
		-3.89219	6.00872	47.65479	57.32385	58.47834	63.35394
		11.75423	14.15204	25.50434	39.73416	59.81865	66.88514
		9.43384	29.84078	29.40868	43.39011	60.45948	64.26337
		3.068832	21.22936	26.32499	24.77543	53.57366	62.17576
		10.63236	22.16027	24.73279	45.7576	55.02466	63.96105
		8.1084	11.87357	13.40813	32.45282	67.66843	67.1134
		12.59584	18.52505	22.2124	31.227	65.99954	66.19546
	5α-DHT + Callindol -5M	30.9672	46.58709	48.61405	45.6486	63.11477	65.33943
		27.45278	44.13721	41.55046	47.39775	62.16369	63.15129
		41.96511	57.2038	60.77866	52.12885	62.12493	64.67117
		25.57433	37.97812	38.62207	35.17338	55.42928	61.49476
		22.84258	32.61992	34.61121	31.57573	58.83561	63.7253
		25.61301	42.90009	53.25885	57.80448	59.25748	62.72245
		22.79887	37.1855	45.99771	51.90998	60.21147	64.8165
		52.86866	68.83825	60.51907	59.38158	60.60724	63.84476
		49.01139	61.87517	74.01457	63.16172	64.69255	65.96137
		38.76412	42.63635	50.15799	53.76928	57.52574	69.6382
		37.20327	53.1987	57.20036	59.60296	60.35298	62.84021

	Log 5α-DHT	-8	-7	-6	-5	-4	-3
22-day pregnant rats	5α-DHT	3.3679	4.50612	13.66806	21.21897	57.4004	62.23839
		-0.65642	16.97687	8.74088	15.80418	63.89503	62.83093
		9.09236	23.44673	10.1016	13.37486	44.33402	61.81806
		-0.35497	18.15748	25.46835	36.76276	49.41174	63.64997
		-1.74926	4.30936	6.26599	4.21672	27.87147	52.64874
		-7.7573	5.34792	8.18114	16.90145	53.62864	66.95464
		-3.4334	3.21204	15.72791	27.21528	39.90439	61.24506
		5.94087	8.44288	17.35859	21.7708	36.02216	60.39771
		-5.04681	11.01277	13.73011	28.0683	40.91461	54.42041
		-6.1301	0.48469	6.63307	24.70792	26.83244	58.88805
		-7.25242	3.65091	17.57565	38.42983	51.86624	69.24811
		-3.00972	8.45968	18.71203	27.31366	47.51938	66.49011
		1.95481	15.34435	24.14284	29.3851	31.89428	47.80929
		11.65257	0.71838	17.21265	8.34463	40.93513	65.49382
		-0.83427	3.58782	8.49563	20.31718	56.02323	67.38843
		7.702403	16.55234	27.88867	25.82554	63.11739	61.61675
		9.72585	11.45891	21.80851	39.43064	47.01052	66.38935
		8.63653	21.11201	29.02733	26.64946	51.37659	63.97592
	5α-DHT + Paxillin -5 M	10.37	21.66519	28.06538	32.46244	48.98118	57.31318
		-1.00391	9.54234	17.09703	21.84271	45.42517	61.38308
		8.6383	14.90384	33.63531	38.28941	54.44789	57.71644
		5.955827	13.82098	23.36206	39.86695	53.77665	60.00269
		10.23517	24.2195	30.437	44.16619	52.44078	50.51353
		-5.89651	13.31746	27.74107	32.45948	52.02384	55.66668
		2.19388	15.62069	21.69157	44.3411	56.7403	59.27137
		39.2372	14.17878	23.21872	43.44926	60.42935	63.52397
		6.70987	17.80015	26.33536	30.6358	58.73788	62.24348
		5.92375	16.83684	24.26828	35.6742	60.06474	59.99095
		1.261966	6.03286	9.84769	5.97183	56.42704	58.35097
		8.63852	16.35451	15.6988	11.09499	56.97805	57.57121
		5.483062	14.43094	18.89212	10.99951	62.59554	63.67848
		11.09151	29.29135	37.88525	35.33302	54.61794	57.7273
		-4.87985	-2.45872	-3.96578	-5.62892	50.01784	53.49511
		-3.99074	6.857341	11.79272	15.20831	55.82779	61.52561
	5α-DHT + NPS -5 M	-0.6802	9.31205	17.42177	25.22678	36.84987	59.13197
		-2.5538	5.361116	21.44363	29.5867	35.81342	48.26561
		3.064645	13.90076	21.76124	23.69608	34.81252	51.01015
		1.471323	16.91917	26.05331	28.28184	42.35546	63.97087
		9.5687	14.467	20.2777	25.6339	42.44423	46.51875
		-14.2723	4.62412	10.90693	5.88378	27.75907	44.33387
		1.111473	12.54569	23.34815	24.20084	37.83988	56.43492
		8.97906	15.20528	22.53774	29.88842	34.93328	60.67661
		5.6889	12.64501	25.60488	30.06939	45.36583	59.53797
		4.64428	14.50792	26.35134	33.54605	43.19986	66.31097
		6.23726	13.76363	26.27448	31.81029	42.598	58.44487
	5α-DHT + Callindol -5 M	-11.8396	1.18312	38.95994	43.29056	48.02972	59.33901
		8.42824	12.00824	39.27366	41.47245	52.56265	66.7876
		4.95786	14.63767	38.03433	41.10598	45.30288	62.95572
		7.81185	10.52229	23.48977	47.22311	48.158	53.95692
		14.03878	26.85678	25.48986	47.51453	55.95254	58.76759
		2.341608	38.77804	34.05362	48.7138	56.99521	67.69085
		11.27232	28.59051	37.07381	43.22515	56.53703	61.07224
		9.22041	33.72381	44.64909	45.45501	56.72278	58.14734
		5.834	17.46752	42.90735	48.18289	56.07621	62.20136
		7.518142	12.0981	33.91148	26.80365	53.99956	55.10747
		24.89595	34.09995	36.63623	45.56791	49.58088	61.48983
		28.46201	39.90198	41.58043	42.90842	58.24426	62.65503
		24.70986	26.48138	31.53722	38.87406	45.81848	53.54177

	Log 5β-DHT	-9	-8	-7	-6	-5	-4
Non-pregnant rats	5β-DHT	-3.70023	7.5413	21.93878	34.40224	51.3322	65.42762
		-8.1633	10.06067	22.97819	35.96335	50.59245	66.35678
		2.436611	15.49607	19.43247	16.88152	57.55571	68.9046
		-3.25715	11.87262	27.1308	37.21998	46.31684	64.44911
		-8.3292	14.60328	25.53124	33.08488	50.39057	66.57987
		-1.93724	15.95504	21.99898	33.3654	51.3757	65.63386
		-5.42013	12.5437	20.7681	35.00558	59.67354	69.41128
		-6.65827	16.83748	29.43047	37.13926	57.1775	63.60165
		0.310816	7.90927	18.98865	28.42126	52.16752	65.77921
		-1.0936	7.01199	18.55233	21.37778	53.64659	68.45351
		-7.4999	14.77249	24.6704	42.3867	54.37507	67.94833
		2.013316	11.66532	22.59825	36.4947	55.77597	62.73521
		3.325814	18.85778	27.05691	38.25581	54.31738	70.63808
		-7.3455	12.4583	22.8663	37.912	54.33778	67.91615
		-2.24591	7.79403	12.14156	30.33774	48.50053	65.33888
		5.909246	12.00419	19.7959	26.11417	42.52935	66.49807
		-2.02524	18.27645	20.76372	32.42288	51.89879	66.2487
		-7.87014	20.452	27.6863	34.0414	50.2346	61.43555
		-2.0875	8.87796	25.575	33.95501	47.24357	66.77871
		-1.9531	12.54616	27.59477	35.74315	43.44033	64.42729
	5β-DHT + Paxilline -5 M	4.09416	7.87791	16.78021	24.39957	55.01336	68.31423
		3.92152	9.1153	12.11576	28.64285	40.81007	65.92506
		1.28034	6.23108	9.5695	17.0004	46.70651	67.89152
		4.596562	10.34207	13.2957	23.369	45.41285	65.78567
		9.83658	10.99665	21.11767	45.09057	59.42039	66.53743
		3.231743	2.731596	12.61102	32.81649	39.83913	68.16862
		6.195396	10.95229	11.1821	31.67953	37.50203	66.45112
		9.08958	17.81165	16.71166	21.11744	35.91868	66.80788
		11.46623	18.3745	11.40049	29.41051	38.17499	64.55682
		2.20088	18.98445	18.64344	24.73016	45.33873	66.12367
		7.55358	12.03812	22.4638	21.58323	59.24884	67.19255
		8.89037	19.29007	11.89825	26.67738	36.04879	64.82153
		9.12273	13.24426	12.42108	5.79266	42.88162	67.82935
		2.75725	3.19682	19.60719	27.2913	37.33735	56.04337
	5β-DHT + NPS -5M	-2.88159	6.86896	13.22449	26.20203	45.95231	66.46067
		6.19581	15.80864	23.10145	20.24541	34.26209	53.52443
		-9.76417	11.08049	24.35287	30.78438	45.77063	65.43226
		-3.11044	9.80981	23.17882	32.15865	52.76738	67.20252
		5.06875	8.30118	26.41884	24.80795	35.46219	65.19489
		6.70878	7.28938	16.5664	26.39546	34.7557	55.10515
		6.210171	18.21042	40.51211	37.12128	40.78568	54.22386
		-2.3898	11.20307	12.88412	20.87192	37.6119	66.31737
		-7.75641	10.90154	18.71744	21.26005	34.34978	67.98054
		8.40979	17.39831	24.28853	24.00134	42.87179	65.7341
		7.41983	15.6858	27.0827	31.79446	40.94504	68.12371
		0.60908	8.6966	15.10535	23.63035	41.90547	65.73787
		7.095614	12.3395	11.44834	19.7403	36.44241	65.55984
		-9.382	9.56945	24.45247	21.17589	36.15394	62.54762
		3.3801	13.54704	25.77785	34.27819	46.25383	52.92302
	5β-DHT + Callindol -5M	5.42365	10.03089	29.40771	31.30515	58.33715	66.03828
		5.68234	14.8648	21.70297	36.42897	50.55478	67.87721
		10.28466	17.33563	25.75337	42.31798	45.2743	53.13657
		12.28736	13.95945	32.89466	34.73862	51.89462	67.67304
		23.59694	18.37067	24.09284	39.00189	46.85896	66.19797
		7.6136	9.01747	31.92777	35.63714	32.20462	66.76064
		17.55101	25.92001	29.27807	30.9758	40.92449	67.75449
		11.85791	17.51008	15.54991	37.37811	44.98866	61.76557

	Log 5β-DHT	-9	-8	-7	-6	-5	-4
22-day pregnant rats	5β-DHT	7.06385	18.24483	28.96179	31.77075	52.18843	55.20654
		5.293839	22.61353	28.18282	21.76425	59.87547	60.94604
		3.569544	5.144506	24.95818	33.83798	49.37951	51.62027
		3.542728	8.493027	28.1391	34.37024	59.05624	58.79446
		2.561642	15.57709	26.64274	36.00384	60.698	62.47071
		1.82721	7.677819	28.17618	37.96701	56.27013	58.10002
		8.639111	18.2627	22.86845	38.3102	50.47051	52.25847
		2.812435	10.44325	28.84619	38.44099	51.31072	55.40682
		2.165981	16.13321	24.91061	32.63641	41.9808	50.29098
		6.380361	14.6519	25.44435	36.11222	52.78031	54.71674
		1.927498	15.38731	28.38206	36.67207	45.51752	53.46431
		3.542728	8.493027	28.1391	36.30629	42.05624	50.79446
		-0.93035	15.15241	17.19528	42.13419	59.58169	53.29205
		13.35338	28.42212	29.21531	47.89871	54.20676	55.51801
		25.2623	37.49501	36.43685	47.27343	59.0393	68.67946
		12.06275	9.630905	12.05315	22.43308	55.59756	59.44163
		10.75339	21.34665	29.00849	35.12692	42.46441	50.32174
		17.82197	25.15829	27.59928	34.79097	47.2678	62.37174
		12.51813	25.24585	33.57863	32.64639	48.28602	67.87272
		19.74091	33.02549	33.8028	38.7723	49.95814	64.01126
	5β-DHT + Paxilline -5 M	-1.3876	-2.02299	14.14928	31.25697	40.34297	56.40065
		3.813499	3.579431	16.5325	32.82955	44.6336	62.23475
		3.455891	6.994351	20.58473	37.98327	40.71102	59.8893
		-1.76271	-0.05467	5.729488	28.38727	42.42857	55.72279
		2.243512	-1.03452	10.78193	13.32589	34.25026	53.00026
		1.353931	5.579023	7.616103	13.72747	39.06124	57.02571
		10.24235	21.7917	24.10841	23.80194	53.27967	64.1488
		19.90672	29.58493	38.65361	49.2383	55.96972	67.45848
		12.33338	16.62341	25.98379	30.56169	57.74493	67.77744
		10.02217	8.55326	20.13687	33.49777	42.48782	57.85918
		10.20383	13.94986	12.5602	29.71903	38.16528	62.02427
		5.792189	17.73162	21.84757	36.33016	50.32863	57.66179
		7.439868	19.86544	21.12451	13.08945	31.06258	66.13469
		14.64141	25.36785	37.72145	30.25186	44.23459	67.11411
	5β-DHT + NPS -5M	4.8214	11.6708	12.6105	13.2151	57.8955	61.3234
		11.1872	19.0941	30.0731	27.8159	62.6137	66.4982
		9.4161	14.1865	23.6523	19.3115	34.5693	57.7201
		11.8806	21.3136	18.84	23.1083	46.2016	54.7718
		0.4476	4.8984	6.5757	6.705	47.6657	60.7919
		-0.7323	5.3899	12.2051	19.4895	47.3805	58.9977
		9.591182	18.80346	21.8222	24.90812	41.28159	55.1908
		9.128828	14.93464	19.10695	23.3927	38.42356	51.5749
		3.817485	14.2875	14.24274	22.84551	54.68884	59.44055
		2.4504	17.78655	20.11734	23.04057	46.63498	56.46313
		6.18299	13.41418	13.7375	29.2142	56.20767	57.64939
		0.726158	4.304178	10.5729	12.94493	58.05514	62.19462
	5β-DHT + Calindol -5M	9.80427	21.38674	30.89687	39.17185	62.51775	65.59572
		9.63502	14.13432	21.49144	15.77864	63.60428	65.97953
		9.3278	15.015	21.61359	31.50057	66.24848	65.08044
		-4.80183	9.3298	11.67046	25.7047	64.08555	65.1193
		3.217639	13.87811	25.22005	31.14826	63.55836	64.68899
		13.47669	14.85744	17.05437	11.20198	64.71101	66.13531
		10.03422	15.66452	27.11468	21.34835	64.60621	64.97848
		6.219192	14.18114	18.12214	19.44771	63.71018	63.69562
		4.41869	10.62342	22.6265	26.20867	47.30187	64.22136
		-3.81448	12.33618	24.29237	34.1559	49.3768	60.34794
		-2.71565	11.34383	17.27276	33.89158	38.31573	57.35269
		6.032959	10.68597	24.4061	44.50218	45.51868	64.95126
		6.29712	13.44297	27.21247	36.45421	39.61725	64.03874
		3.70477	18.20867	26.38369	42.38198	57.06076	63.51491
		2.1688	7.67475	19.78662	38.52626	53.88766	55.11255
		8.08227	18.00594	25.04667	40.6723	45.9669	65.28293

	CaCl2 (mM)	0.47712	1	1.47712	1.77815	1.95424	2.07918
Non-pregnant rats	CaCl2 alone	55.3759	136.371	600.146	1300.18	1813.76	1872.9
		73.4802	174.472	787.206	1621.41	2483.94	2605.23
		81.3462	255.854	836.874	1508.24	1830.83	1357.17
		122.978	227.352	719.736	1557.19	2154.28	2265.7
		27.383	80.3655	361.157	708.826	1093.72	1170
		82.5732	144.453	349.881	698.06	1028.03	1022.6
		49.9302	382.987	1122.6	1720.35	1992.47	2058.4
		44.2745	196.393	675.207	1111.46	1370.82	1477.07
		211.404	551.127	1225.41	1776.32	2151.21	2320.56
		108.729	364.411	1261.65	1993.74	2153.63	2652.3
		174.626	204.471	788.642	1008.09	938.028	1144.63
		163.364	373.439	876.683	1217.63	1307.15	1400.79
		324.234	742.331	2261.22	3830.45	4172.94	4019.05
		263.451	1112.81	2682.48	3875.52	4183.95	4279.28
		112.84	388.445	1214.64	1585.19	1281.89	2477.81
		88.6882	394.29	1069.33	2101.06	2647.63	3265.54
		142.802	617.73	1622.68	2429.46	2621.41	2783.01
		108.903	601.222	1983.65	3289.02	3649.3	3894.79
		152.668	1018.87	3064.11	4356.21	4422.11	4121.08
		33.9623	625.284	2116.08	3089.89	3222.71	4346.26
		40.8776	248.462	965.586	1311.58	1231.05	1769.3
		59.4966	649.487	1964.28	2852.31	3305.38	3467.3
		120.765	502.163	1365.71	2081.56	2415.32	2580.29
	CaCl2 + 5α-DHT -4M	106.261	159.823	185.788	194.938	245.563	253.215
		106.261	159.823	185.788	194.938	245.563	253.215
		121.028	177.468	190.285	216.09	229.09	240.031
		97.5568	104.256	149.627	189.617	283.446	286.435
		32.3325	27.126	76.7616	214.922	341.876	361.537
		99.1634	123.518	191.635	193.116	201.591	253.466
		91.7586	110.746	114.828	250.118	306.422	333.107
		96.7669	104.072	108.499	274.734	321.701	286.6
		80.5157	93.5831	102.936	255.292	364.024	380.849
		101.485	112.291	120.189	277.725	364.514	343.069
		69.0262	90.6021	163.822	337.73	366.595	373.524
		97.5685	101	108.993	195.242	243.887	243.503
		97.9075	166.037	180.979	196.743	201.795	204.539
	$CaCl_2$+5β-DHT 10^{-4}M	59.2876	69.8258	82.4382	93.8242	98.1525	116.514
		48.3473	54.1635	58.4463	73.0463	84.0316	120.358
		53.2421	66.3924	71.7068	74.0346	78.0402	100.961
		59.0731	74.5944	91.1233	113.047	121.836	141.344
		54.1004	67.5273	79.2218	87.2128	93.1581	103.237
		43.022	47.7905	53.3658	73.2006	78.6709	91.1975
		51.8109	68.5917	76.8037	87.2751	92.468	101.023
		56.2558	65.9235	68.518	74.0732	78.9878	92.7963
		36.4706	54.8917	63.075	66.4067	81.8669	100.119
		50.2975	55.2862	61.5555	75.805	89.721	103.477
		48.6867	55.3474	70.5901	92.5153	99.6264	107.807
		46.9833	67.2151	75.5691	84.4264	99.7501	115.366
	CaCl2 + Nifedipine -7M	51.3931	56.2704	62.6268	64.4118	69.1686	80.8934
		57.0699	63.1404	70.2431	67.3227	82.533	91.5189
		57.8278	61.7307	67.845	82.2136	85.4976	95.1134
		51.1807	57.9419	74.4618	63.4576	77.794	84.9549
		26.3831	36.1727	41.5166	43.1127	47.4797	55.1824
		39.8848	49.6359	52.5064	57.3036	61.6032	71.7299
		38.0145	41.3087	43.4418	50.3635	57.0366	60.8708
		33.2899	33.1526	73.1774	79.9749	86.72	98.0239
		35.2803	29.2265	64.2497	59.4938	46.1972	51.6754
		41.4577	50.0774	55.5172	63.2927	75.8786	82.1921
		40.3751	46.5607	66.9714	74.7317	77.5662	88.1058

	**CaCl2 (mM)**	0.477121	1	1.477121	1.778151	1.954243	2.079181
**22-day pregnant**	**CaCl2 alone**	61.81477	98.59399	230.7045	1303.253	1952.144	2264.988
		93.85204	116.3227	218.6773	736.7116	1009.6	1203.8
		90.28158	115.6119	419.9938	1360.696	1991.65	2254.556
		93.63932	142.1238	445.7577	2253.015	2239.989	2597.307
		91.88277	116.1287	466.1699	1911.263	2479.066	2931.1
		63.20154	117.8529	298.4547	1373.626	2477.638	2630.582
		40.3877	50.53564	205.462	998.8341	1329.385	1353.573
		80.35555	107.6421	326.9962	910.9033	1478.546	1655.807
		156.992	412.8948	1492.353	1928.727	2350.564	3127.57
		144.4214	481.6186	1887.62	2840.116	3171.105	3662.488
		181.4111	365.7265	426.5359	1684.814	2089.55	2506.89
		110.9638	344.9182	489.2314	1031.343	1100.668	1580.613
		173.274	429.251	798.2461	1303.101	1900.305	2467.887
		287.8145	909.6735	1684.681	2539.718	3196.594	3814.371
		68.82541	334.4774	1057.284	1873.094	2771.315	3742.399
		52.70062	217.3223	463.6465	1218.556	1550.175	2066.781
		109.0979	168.8072	575.0268	1110.543	1730.341	2185.713
		146.0641	463.4581	1055.596	1958.932	2613.467	3407.719
		131.142	176.6238	478.3217	1001.54	1664.048	2419.791
		219.2051	209.0754	408.818	1014.934	1700.647	2308.909
		289.9678	350.1671	673.9102	1257.068	1953.467	2639.459
		309.763	419.9224	469.0142	775.9944	1260.722	1847.685
		165.3555	271.6766	604.7228	1369.815	2444.04	3071.593
		121.1357	220.6019	973.6663	2954.41	4894.301	5710.32
		166.9926	294.6174	559.3166	1062.102	1606.951	2183.508
		906.9147	1132.075	597.4424	1301.761	3255.048	5413.535
		125.2895	161.3005	302.3589	486.6161	671.1481	832.371
		31.76831	101.7942	388.8821	1011.67	1675.216	2031.943
		52.19241	115.0777	474.439	1167.238	1472.419	2054.919
		66.34601	103.8568	336.3607	648.2966	957.8568	1346.066
	**CaCl2 + 5α-DHT -4M**	44.14679	67.69887	89.36893	122.9093	146.4146	175.3435
		62.4682	77.5403	91.19639	123.7158	153.4579	171.3833
		70.47421	84.04705	93.9864	106.9462	119.1117	134.1352
		90.552	117.5772	136.9418	159.7564	175.4019	191.841
		94.5864	121.6162	142.521	166.0484	187.8364	215.6225
		95.5251	117.1214	133.0494	157.8041	173.8578	191.5298
		119.0263	149.1225	177.1226	214.7583	243.8656	275.7657
		124.9115	135.3855	152.8859	177.9654	210.3687	226.1867
		122.0998	137.8057	156.9709	170.2536	186.4512	213.9799
		58.87979	76.76054	96.0127	121.5141	142.1351	177.576
		96.40783	107.2083	126.479	148.1095	161.2524	189.147
		124.6682	143.7167	160.1323	189.9656	210.9007	251.6173
	**$CaCl_2$+5β-DHT 10^{-4}M**	23.75587	26.99435	32.64538	41.26421	44.79706	47.20163
		19.34049	26.56362	28.07882	32.36339	45.75704	53.62918
		28.261	43.3331	46.98919	58.50864	69.2507	87.1761
		21.56142	25.62743	33.23803	37.6499	39.8897	49.1981
		18.6403	37.6888	54.1044	83.9377	104.8728	145.5894
		14.41137	18.43247	20.47674	32.47244	47.38089	53.19154
		19.3448	25.37	34.7346	57.5492	73.1947	89.6338
		53.85189	71.73264	90.9848	46.4862	57.1072	72.5481
		26.26701	29.83985	39.7792	52.739	64.9045	69.928
		28.3687	38.8427	56.3431	81.4226	84.8259	99.6439
		32.4835	42.57973	50.5798	58.2155	67.3228	79.2229
		22.12144	31.19749	37.10375	40.00454	43.26963	49.403
		23.1292	30.159	41.0638	63.5912	76.3792	84.1653
		26.1277	41.8336	60.9988	74.2815	90.4791	118.0078
		18.75957	24.29085	27.81046	34.67031	32.94457	46.35389
		26.37993	31.1804	40.4511	62.0816	75.2245	83.1191
		22.35641	31.88331	35.02368	42.68449	52.15263	61.46003
		16.46721	20.83919	26.82108	30.83896	38.74338	42.49126

	CaCl2 + Nifedipine -7M	19.0024	22.34459	25.75384	30.48281	34.60761	38.57207
		24.05853	28.49084	34.12299	39.72711	45.14326	52.63187
		20.68569	25.19927	29.17812	35.90865	41.52692	49.76452
		13.13152	18.13513	23.57698	29.71798	35.93257	41.9076
		22.87969	29.17661	37.54873	43.88557	49.07467	56.84555
		19.34652	25.17704	33.42287	41.85051	49.30937	56.05468
		22.97252	28.06895	32.30049	37.45505	41.34231	45.38583
		22.76669	28.53819	34.35646	40.72616	47.37443	53.64013

NP-Control	5α-DHT10^{-4}M	5β-DHT 10^{-5}M	P-control	5α-DHT 10^{-4}M	5β-DHT10^{-5}M
2.051975	3.129417	3.00791	2.64215	4.387879	3.717113
2.614873	3.528653	3.609244	2.777296	3.764227	2.882684
1.865995	4.727602	3.37739	2.361941	3.533613	2.516924
2.495846	3.815062	3.581967	2.468569	3.864656	3.37243
2.603715	4.735041	4.527984	2.3731	3.65388	4.075433
2.46609	4.049396	4.386639	2.349542	4.125028	4.132467
2.106529			2.446252		
2.061894			2.355742		
			2.64215		

NP: non-pregnant
P: 22-day pregnant rats

NP-control	5α-DHT10^{-4}M	5α-DHT+L-NAME	5β-DHT 10^{-5}M	5β-DHT+L-NAME		P-control	5α-DHT 10^{-4}M	5α-DHT+L-NAME	5β-DHT10^{-5}M	5β-DHT +L-NAME
0.000318	0.000333	0.000283	0.000317	0.000234		0.000231	0.000309	0.000314	0.000347	0.000329
0.000279	0.000284	0.000206	0.000296	0.000276		0.000389	0.000316	0.000247	0.000355	0.000286
0.000316	0.00033	0.000288	0.000374	0.000232		0.000308	0.000326	0.000416	0.000395	0.000334
0.000278	0.000364	0.000211	0.000427	0.000272		0.000239	0.000321	0.000356	0.000326	0.000283
0.000344	0.000338	0.000285	0.000319	0.00023		0.000365	0.000308	0.000401	0.000284	
0.000325	0.000287		0.000294	0.000273		0.000362	0.000252	0.000319	0.000415	
0.00039	0.00034		0.000376			0.000337	0.000319	0.000248	0.000342	
0.000299	0.00037		0.000419			0.000349	0.000393			
	0.000342									
	0.000293									

NP: non-pregnant
P: 22-day pregnant rats

Time (min)	5α-DHT non-pregnant rats						5α-DHT 22-day pregnant rats			
0	0	0	0	0	0		0	0	0	0
5	97.0389	87.5525	81.7674	87.3237	82.1694		65.2069	96.9263	74.9781	125.8788
15	117.755	127.862	96.6974	109.7272	115.7357		134.5375	116.8294	143.6024	149.0022
30	102.4177	90.9742	76.0027	92.7013	74.7468		92.2718	75.1814	107.3014	99.8312
60	83.2012	68.4914	53.0053	73.1311	48.7194		59.9727	57.6115	77.6476	80.6186
120	66.1997	51.9649	35.4529	51.6726	36.497		42.7363	34.806	53.3296	64.536
240	49.9513	34.668	19.1964	30.656	23.1554		26.8855	17.1063	41.0927	44.1286
480	10.9362	5.0549	7.6694	9.4905	3.5953		0.9757	7.35	14.2936	31.2303

Time (min)	5β-DHT-non-pregnant rats						5β-DHT 22-day-pregnant rats				
0	0	0	0	0	0		0	0	0	0	0
5	88.965	105.563	82.0976	123.4066	88.1432		91.1571	97.522	82.664	101.2991	82.8007
15	117.394	152.7864	117.2125	176.3068	112.14		130.2021	123.6367	125.4162	126.2091	127.3805
30	85.424	93.5155	67.9449	121.1167	79.904		84.7499	76.235	83.2898	75.3718	89.7756
60	70.697	67.8247	47.1911	97.4519	64.0967		77.7993	67.5867	58.6619	55.2468	79.1994
120	49.963	46.8467	27.9092	73.8234	40.1234		57.4081	49.7782	41.8082	38.0334	61.8456
240	31.022	27.6797	17.7657	50.8483	19.3548		31.2712	29.889	25.9819	28.1947	28.2756
480	10.345	17.4507	10.751	20.0436	11.0935		6.9944	6.6691	4.1179	5.6185	7.3525

DHT levels before and after 30 min

	NP-control	3 mg/kg	10 mg/kg	30 mg/kg	100 mg/kg	300 mg/kg		P-control	3 mg/kg	10 mg/kg	30 mg/kg	100 mg/kg	300 mg/kg
	131.962	145.6183	219.2654	240.3118	240.015	288.5876		163.7513	167.4426	261.983	233.4121	267.474	334.0255
	155.2743	129.2768	228.2337	226.8039	250.3476	290.4864		159.9354	171.1011	255.6303	256.6291	265.0305	302.3733
5α-DHT	150.2556	161.0593	220.7352	242.5995	242.4676	281.8766		190.3381	182.8836	228.449	253.3645	285.6404	350.7841
	142.4679	146.461	218.6384	217.5005	278.8278	299.2408		183.4712	178.2853	287.7476	256.5276	253.5524	315.9572
	98.98362	114.8247	230.4352	231.949	252.9542	293.0308		138.2338	186.649	209.6988	249.8679	267.7927	325.4735
	79.58661							171.1331					

	NP-control	3 mg/kg	10 mg/kg	30 mg/kg	100 mg/kg	300 mg/kg		P-control	3 mg/kg	10 mg/kg	30 mg/kg	100 mg/kg	300 mg/kg
	131.962	165.2564	208.428	207.0037	221.5333	215.2863		163.7513	182.172	230.834	267.5225	244.2721	286.4086
	155.2743	148.9149	224.4368	235.8982	235.7817	225.5369		159.9354	165.8305	236.0382	209.3568	222.635	277.5581
	150.2556	180.6974	210.8621	214.5921	227.7927	352.0406		190.3381	197.613	221.2488	261.9264	254.62	300.7484
5β-DHT	142.4679	166.0991	236.8694	180.4521	224.203	230.6497		183.4712	183.0147	239.9457	215.3005	270.6528	300.7683
	98.98362	134.4628	201.8288	232.625	237.7486	348.9745		138.2338	151.3784	220.4579	213.8932	239.8846	352.0116
	79.58661	160.0071		250.4896	220.5892			171.1331	176.9227		243.6287		

NP: Non-pregnant

P: 22-day pregnant

In vivo action

	Log dose mg/kg	5α-DHT alone									5α-DHT+ Flut. 100 mg/kg								
Non-pregnant rats	0.477121255	32.86441	26.95081	33.75019	32.63889	28.16378	13.79799	13.6675	22.48867		31.4944	29.0722	36.95138	28.6734	22.4669	17.4883	25.6477	12.5861	
	1	32.88057	23.08005	49.81885	44.26687	27.84469	22.60968	28.4872	34.7863		45.8258	38.3992	42.9588	32.0181	23.4784	42.021	34.7891	36.6734	
	1.477121255	55.58546	23.3267	47.40289	37.45755	40.80697	30.82355	42.5448	50.7199		42.6428	38.5548	52.4773	33.4595	41.3301	33.3693	51.4089	58.7472	
	2	51.15049	35.608	36.27707	30.75953	46.11197	59.97306	74.67237	69.58483		53.4335	59.6831	64.4533	55.2551	48.9187	69.6477	51.8821	38.4399	31.6958
	2.477121255	55.18862	59.11639	50.87143	58.67673	78.4837	83.7395	60.95442	88.74789		67.8996	81.3309	87.4668	61.8905	58.5451	65.3686	59.9089	77.8964	

	Log dose (mg/kg)	5α-DHT alone									5α-DHT+ Flut. 100 mg/kg							
Pregnant rats	0.477121255	6.457725	13.00579	7.999628	7.276659	10.02368	5.8669	9.4869	6.0825		5.81683	3.2746	7.66031	9.194532	7.8414	5.5626	4.9639	8.8221
	1	16.32847	15.80788	16.14717	9.789656	5.360948	10.9752	12.7938	8.6264		6.1789	27.3647	6.6893	9.4876	5.9128	12.9385	3.9565	7.8928
	1.477121255	9.940428	10.59629	38.84039	24.77191	29.42471	28.6253	34.8821	22.8604		10.4876	18.1765	27.9187	25.3468	20.7301	35.8269	15.8586	38.7263
	2	52.94469	49.51006	36.47827	42.27888	41.6882	58.8633	31.3296	47.086		53.6789	38.9123	27.1894	41.8505	41.8355	48.8938	36.7969	31.5308
	2.477121255	54.50088	40.49313	33.42461	50.15647	57.10933	43.535	40.8692	58.9639		43.8123	42.7381	47.4123	38.7325	33.0862	52.08569	35.323	49.6535

	Log dose mg/kg	5β-DHT alone									5β-DHT + Flut. 100 mg/kg							
Non-pregnant rats	0.477121255	10.49576	38.2141	15.71013	10.89072	15.22713	12.82688	13.5669	18.9199		18.6739	27.91808	21.64212	17.45738	22.2832	31.6289	27.4097	20.2979
	1	17.25307	30.56065	34.55941	32.17387	33.36733	24.6448	42.2826	21.4582		23.7193	25.53826	29.90235	27.08632	34.4869	30.6085	19.727	21.1454
	1.477121255	38.88876	32.98294	55.93479	50.75172	49.01158	25.8554	49.4228	42.0496		45.38312	38.18931	42.03104	47.22465	45.0397	42.4301	56.2896	55.4089
	2	63.44663	62.91625	66.71691	51.18103	56.91933	45.74837	55.52587	55.8255		71.91021	69.5391	65.41805	48.4292	63.70632	62.05931	73.5083	68.8698
	2.477121255	75.44746	74.28881	73.89108	71.19042	81.9187	78.7344	71.8343	73.8507		76.20314	81.29773	77.14287	78.16474	69.44073	82.1868	79.8565	76.43308

	Log dose mg/kg	5β-DHT alone										5β-DHT + Flut. 100 mg/kg								
Pregnant rats	0.477121255	20.54277	17.07485	14.59579	22.3453	25.6864	22.8265	12.4459				25.73527	18.92283	12.64725	7.582871	24.2285	37.7045	18.442	11.5053	
	1	29.78665	36.39632	30.45295	32.84718	30.49313	20.8464	36.4536	22.8863	18.7826		33.87821	39.85213	36.62834	31.43897	33.87821	48.3315	34.0845	20.4231	
	1.477121255	38.0738	49.60144	32.83844	35.5074	39.2062	42.0461	32.1868	49.88371			35.27357	34.76581	48.4317	41.36827	45.23417	42.38663	39.6424	30.08632	28.1638
	2	52.94469	49.51006	42.47827	42.27888	55.6849	42.7275	57.6386	36.6832			51.74849	58.25347	43.44567	55.29719	53.72872	66.2628	52.3495	62.1898	47.6426
	2.477121255	54.50088	59.45769	54.72319	63.2839	53.42461	53.7086	59.9863	47.8632			66.94157	51.22468	63.78395	54.83947	59.34562	69.4255	55.4996	53.8836	50.8638
